# Two MicroRNAs Are Sufficient for Embryonic Patterning in *C. elegans*

**DOI:** 10.1016/j.cub.2020.09.066

**Published:** 2020-12-21

**Authors:** Philipp J. Dexheimer, Jingkui Wang, Luisa Cochella

**Affiliations:** 1 Research Institute of Molecular Pathology (IMP), Vienna BioCenter (VBC), Campus-Vienna-Biocenter 1, 1030 Vienna, Austria

**Keywords:** microRNA, Drosha, Pasha, DGCR8, mirtron, embryogenesis, development, *C. elegans*

## Abstract

MicroRNAs (miRNAs) are a class of post-transcriptional repressors with diverse roles in animal development and physiology [1]. The Microprocessor complex, composed of Drosha and Pasha/DGCR8, is necessary for the biogenesis of all canonical miRNAs and essential for the early stages of animal embryogenesis [2, 3, 4, 5, 6, 7, 8]. However, the cause for this requirement is largely unknown. Animals often express hundreds of miRNAs, and it remains unclear whether the Microprocessor is required to produce one or few essential miRNAs or many individually non-essential miRNAs. Additionally, both Drosha and Pasha/DGCR8 bind and cleave a variety of non-miRNA substrates [9, 10, 11, 12, 13, 14, 15], and it is unknown whether these activities account for the Microprocessor’s essential requirement. To distinguish between these possibilities, we developed a system in *C. elegans* to stringently deplete embryos of Microprocessor activity. Using a combination of auxin-inducible degradation (AID) and RNA interference (RNAi), we achieved Drosha and Pasha/DGCR8 depletion starting in the maternal germline, resulting in Microprocessor and miRNA-depleted embryos, which fail to undergo morphogenesis or form organs. Using a Microprocessor-bypass strategy, we show that this early embryonic arrest is rescued by the addition of just two miRNAs, one miR-35 and one miR-51 family member, resulting in morphologically normal larvae. Thus, just two out of ∼150 canonical miRNAs are sufficient for morphogenesis and organogenesis, and the processing of these miRNAs accounts for the essential requirement for Drosha and Pasha/DGCR8 during the early stages of *C. elegans* embryonic development.

**Video Abstract:**

## Highlights

• Depletion of Drosha and Pasha results in embryos that fail to undergo morphogenesis
• The mirtron pathway enables expression of miRNAs in the absence of Drosha and Pasha
• Two miRNAs are sufficient to rescue embryogenesis in the absence of Drosha and Pasha
• miR-35 and miR-51 play an unexplored, likely conserved role in animal development

## Graphical Abstract

**Figure:**
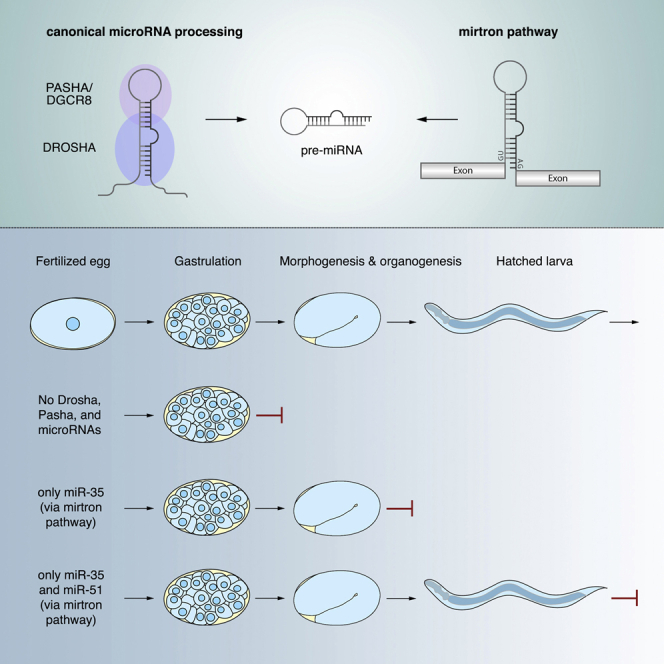

## Teaser

Dexheimer et al. show that depletion of maternal and zygotic Drosha and Pasha in *C. elegans* embryos causes failure to undergo morphogenesis. This can be overcome by re-introducing two miRNAs, miR-35 and miR-51, resulting in morphologically normal larvae. These conserved miRNAs account for the essential embryonic function of Drosha and Pasha.

## Results

### The Microprocessor Is Necessary for Morphogenesis and Organogenesis in *C. elegans*

MicroRNAs (miRNAs) are short non-coding RNAs that act with Argonaute proteins to elicit translational repression and decay of specific target mRNAs [1]. miRNAs are excised from longer genome-encoded transcripts by the Microprocessor, a complex of the endonuclease Drosha and its partner Pasha/DGCR8, and further processed by another endonuclease, Dicer [1]. Loss of the miRNA processing machinery or the Argonaute effector proteins results in early developmental lethality in every animal species studied [16], but the cause for this strict requirement remains unclear. In zebrafish, the abundant miR-430 accounts for a large fraction of the embryonic defects observed upon loss of Dicer [17], acting at least in part by clearing maternal mRNAs [18]. However, miR-430 is only present in vertebrates, and in zebrafish in particular, it forms an unusual cluster with >50 genomic copies [19]. In other animals, the reasons for the essentiality of the miRNA pathway during early embryonic development remain unknown.

**Figure 1:**
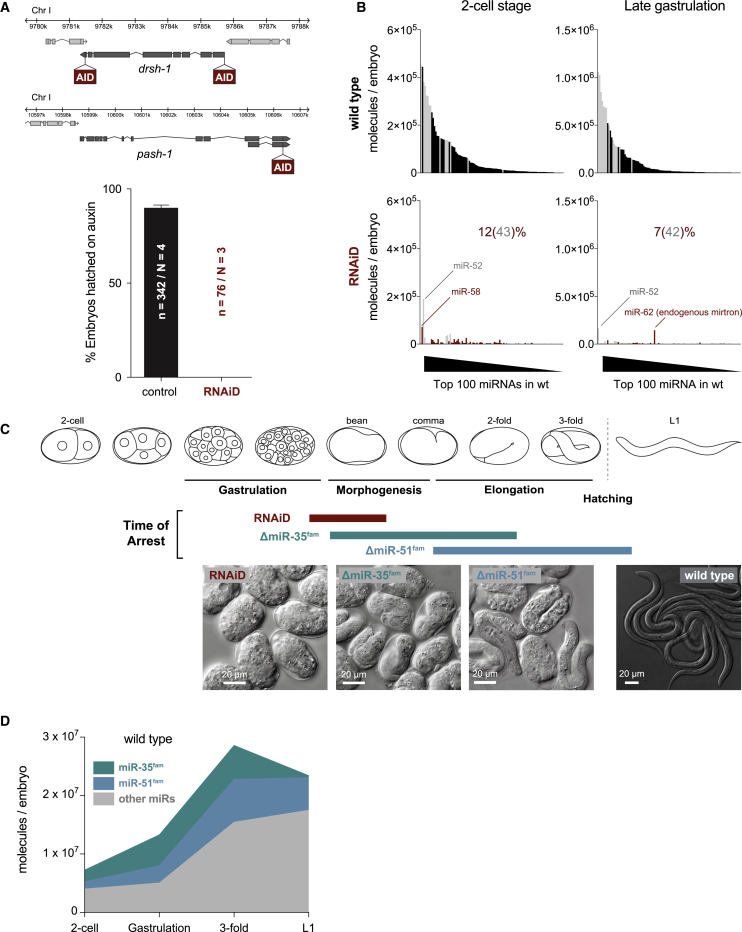
miRNAs Are Essential for Morphogenesis and Organogenesis (A) Schematic of the genetic setup to deplete the Microprocessor via the AID system and hatching rate of control (expressing only TIR1) or Microprocessor-depleted embryos by RNAiD. n, number of embryos scored; N, number of independent experiments. Error bars represent the standard error of the proportion. (B) Absolute miRNA abundance in WT and RNAiD embryos at the 2-cell stage and at the end of gastrulation as determined by quantitative small RNA sequencing. The 100 most abundant miRNAs in WT embryos at each stage are shown. Remaining miRNAs of the miR-35 and miR-51 families are depicted in gray. The percentage of miRNA molecules remaining relative to WT is indicated, with relative contributions of miR-35 and miR-51 families combined noted in brackets. Note that the samples analyzed here are the same as in Figures S2F, S2G, 4A, and S4A. (C) Schematic of the predominant time of arrest and representative images of embryos of the indicated genotypes. (D) Absolute miRNA abundance in developing WT embryos as measured by small RNA-seq, highlighting the proportion of miR-35 and miR-51 families. See also Figure S1.

To investigate the role of the Microprocessor during embryogenesis of the nematode *C. elegans*, we developed a conditional approach for stringent removal of Drosha and Pasha/DGCR8 and thus miRNAs. Microprocessor depletion in embryos is generally challenging, given the maternal contribution of mRNA and protein. In *C. elegans* in particular, the function of the Microprocessor has not been studied in embryos because homozygous Drosha (*drsh-1*) or Pasha/DGCR8 (*pash-1*) mutants derived from heterozygous mothers carry enough maternal RNA and/or protein to develop into adults, albeit sterile [2, 3]. To overcome this, we took advantage of the *Arabidopsis* auxin inducible degradation (AID) system [20]: we tagged endogenous Drosha (both at the N and C termini) and Pasha/DGCR8 (at the C terminus) with the TIR1 ubiquitin ligase-recognition peptide and expressed TIR1 in both germline and soma to clear maternal and zygotic tagged proteins (Figures 1A and S1A). To further reduce Microprocessor levels, we simultaneously triggered systemic RNAi against *pash-1* by feeding (Figure S1B). To ensure elimination of maternal Microprocessor and its products, mothers were grown on auxin and RNAi-eliciting bacteria for 20 h before harvesting just-fertilized embryos (2-cell stage). This treatment caused an increase in unfertilized oocytes and a concomitant reduction in the number of embryos produced, as expected based on the described roles for miRNAs in germline function [2, 3, 21, 22, 23]. However, we could still harvest enough 2-cell embryos and follow their development. Whereas depletion of either PASH-1 or DRSH-1 alone was sufficient to induce penetrant embryonic lethality (Figure S1C), simultaneous removal of both proteins and *pash-1* mRNA resulted in the earliest, most homogeneous arrest phenotype (Figures S1C–S1E). We refer to this combined AID and RNAi manipulation as RNAiD. This system enabled examination of *C. elegans* embryos that were largely depleted of maternal and zygotic miRNAs (Figure 1B). 100% of these embryos arrest uniformly at the end of gastrulation/onset of morphogenesis, lacking distinguishable internal structure other than the intestine primordium, and eventually die (Figures 1A, 1C, S1D, and S1E).

To deplete the Microprocessor by independent means, we used *pash-1(mj100)*, a temperature-sensitive allele of Pasha/DGCR8 [24]. Shifting mothers to the restrictive temperature 7 h before harvesting 2-cell embryos (the maximum possible before the mothers visibly deteriorate) also resulted in fully penetrant embryonic lethality (Figure S1F). However, the timing of arrest was variable (Figures S1D and S1E), consistent with the observation that an average of 35% of maternal miRNA content remained at the 2-cell stage (Figure S1G). Thus, although the *pash-1(ts)* allele is efficient for removing miRNAs from gastrulation onward, RNAiD results in stringent miRNA depletion from oogenesis up to the time of embryonic arrest.

In *C. elegans,* no individual miRNA is known to be essential for embryogenesis, but two families with multiple, redundant members each, miR-35^fam^ and miR-51^fam^, are necessary at two distinct time points [25, 26, 27]. Deletion of all eight members of the miR-35^fam^ (miR-35-42) causes arrest after completing gastrulation during early morphogenesis, -whereas animals lacking the six miR-51^fam^ members (miR-51-56) have later elongation and organogenesis defects and arrest as late embryos or malformed larvae (Figures 1C, S1D, and S1E). These two families constitute ∼50% of the early embryo miRNA content (Figure 1D), and they are broadly if not ubiquitously expressed during embryogenesis [26, 27]. The remaining >130 miRNAs are individually much less abundant and tend to be expressed in cell-type-specific manner [16, 28, 29].

The effect of RNAiD was more severe than the arrest phenotype of animals lacking the early-acting miR-35^fam^ (Figures 1C, S1D, and S1E), raising the possibility that other miRNAs could be necessary for earlier stages of embryogenesis. Alternatively, non-miRNA-related activities of Drosha and Pasha/DGCR8 could be responsible for the more severe phenotype upon RNAiD. Moreover, it is possible that the combined loss of miR-35^fam^ and miR-51^fam^ accounts for the observed defects in embryogenesis in the absence of the Microprocessor.

### The Mirtron Pathway Can Be Used to Produce Microprocessor-Independent miRNAs *In Vivo*

**Figure 2:**
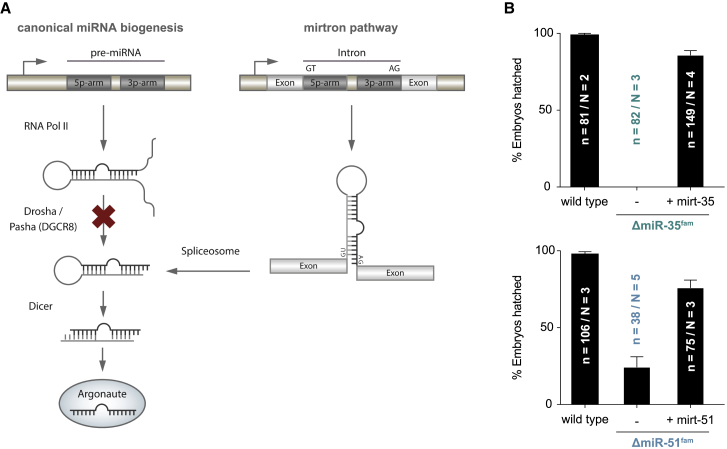
The Mirtron Pathway Can Substitute for Abundant Endogenous miRNAs (A) Schematic of the experimental strategy to test for miRNA sufficiency: a block in miRNA biogenesis at the Microprocessor level is bypassed by splicing during the process of mirtron biogenesis. (B) Hatching rates of miR-35^fam^ and miR-51^fam^ mutant embryos with or without mirt-35 or mirt-51, respectively. Error bars represent the standard error of the proportion. See also Figure S2.

To distinguish between the possibilities raised above, we asked whether miR-35^fam^ and miR-51^fam^ are sufficient for embryogenesis in the absence of the Microprocessor and all other miRNAs. As every individual member of the miR-35 or miR-51 family can rescue the complete deletion of the respective family [26], we reasoned that we could re-introduce one member of each family into Microprocessor-depleted animals to test their sufficiency. Previous approaches to introduce miRNAs in a Microprocessor-independent manner relied on injection of processed RNA duplexes [17]. Instead, we developed a transgenic strategy for Microprocessor-independent miRNA delivery, which recapitulates the spatiotemporal specificity of expression of re-introduced miRNAs. Specifically, we designed miR-35^fam^ and miR-51^fam^ mirtrons, which are processed by the spliceosome and are subsequently cleaved by Dicer to produce mature miRNAs (Figures 2A and S2) [30, 31].

Mirtron-35 and mirtron-51 (mirt-35/51) were expressed under their respective endogenous promoters. Each mirtron was able to largely rescue embryonic lethality of the corresponding miRNA family deletion (to 85% and 75%, respectively; Figures 2B and S2E). In contrast, a mirt-35 variant with a mutated seed sequence failed to overcome lethality caused by deletion of miR-35^fam^ (Figure S2E), ruling out a non-specific rescue by mirt-35 due to its sheer abundance and reported interaction of miR-35^fam^ with other small RNA pathways [32].

Small RNA sequencing from animals expressing the mirtrons in Microprocessor-depleted backgrounds revealed that each mirtron was processed accurately into a mature miRNA with the expected 5′ end, preserving the family seed sequence and thus target specificity (Figure S2F) [33]. Both mirtrons displayed heterogeneity at the 3′ end: 25%–45% of mirt-35 had non-templated addition of a single nucleotide, uridine in >90% of cases, consistent with the described tailing of mirtrons in *Drosophila* [34, 35], and 90%–95% of mirt-51 was trimmed by 1 nt, reducing its length from 24 to 23 nt. The mirtrons were practically undetectable in 2-cell embryos, consistent with commonly observed transgene silencing in the *C. elegans* germline. However, correctly processed mirtrons reached levels comparable to those of the respective families at gastrulation and later time points (Figure S2G). This establishes the mirtron pathway as an efficient tool to express Microprocessor-independent miRNAs that substitute for abundant, endogenous miRNAs.

### Two miRNAs Rescue Morphogenesis and Organogenesis in Microprocessor-Depleted Embryos

**Figure 3:**
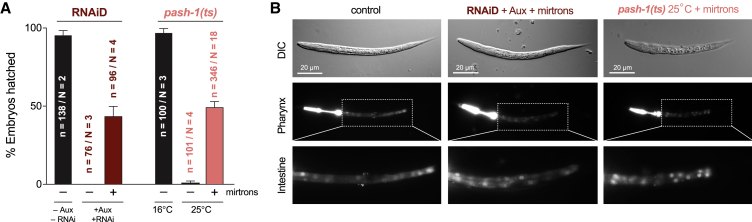
Two miRNAs Are Sufficient for Embryonic Patterning in the Absence of Drosha and Pasha (A) Hatching rates of RNAiD or *pash-1(ts)* Microprocessor-depleted animals, with or without mirt-35 and mirt-51. Error bars represent the standard error of the proportion. (B) Representative images of mirtron-rescued L1 larvae. Shown are differential interference contrast (DIC)/Nomarski images, fluorescently labeled pharynx (*myo-2*^*prom*^*::mCherry*), and intestine (*elt-2*^*prom*^*::NLS-dsRed*). For comparison to arrested embryos without mirtrons, see Figures 1A and S1D. See also Figure S3.

Simultaneous expression of mirt-35 and mirt-51 restored the ability of the RNAiD and *pash-1(ts)* Microprocessor-deficient embryos to produce morphologically normal larvae in ∼50% of cases (Figure 3), out of a theoretical maximum of ∼60%, given the imperfect rescue of each of the mirtron transgenes (Figure 2B). The remaining ∼50% of embryos showed variable levels of rescue, with ∼70% reaching later stages of development, albeit with morphogenesis defects (Figure S3A). Both mirtrons were necessary to rescue embryogenesis up to successful hatching in RNAiD-treated animals. However, in the *pash-1(ts)* background, in which a higher fraction of maternal miR-35^fam^ and miR-51^fam^ members persists in early embryos (Figure S1G), each mirtron provided partial rescue on its own (Figure S3B). Consistent with the earlier role of miR-35^fam^, RNAiD embryos expressing just mirt-35 overcame the early arrest phenotype but failed later during elongation predominantly as malformed 2- to 3-fold embryos (Figures S3C and S3D). Notably, embryos expressing only mirt-51 developed a little further than RNAiD embryos without mirtrons. This suggests that, in addition to a role at later stages of embryogenesis, miR-51^fam^ has a contribution early on, which can be observed in the absence of other miRNAs. We thus conclude that the more severe phenotype of RNAiD relative to miR-35^fam^ mutants is due to the action of other miRNAs, primarily of miR-51^fam^. In addition, RNAiD embryos expressing mirt-51 together with the seed mutant version of mirt-35 looked indistinguishable from embryos expressing mirt-51 alone (Figures S3C and S3D). These results indicate that embryogenesis requires the activity of a miR-35 and a miR-51 family member and not any abundant miRNA that might either balance other small RNA pathways, prevent non-specific loading of Argonaute proteins, or act through distinct indirect mechanisms [32].

Mirtron-rescued larvae formed all major organs and appeared morphologically normal, although they were slightly shorter and wider (Figures 3B and S3E–S3G), likely due to the lack of miR-58^fam^ [26]. They also crawled and fed, indicating that the production of these two miRNAs accounts for the majority of the embryogenesis defects observed in Microprocessor-deficient *C. elegans*. However, rescued larvae rarely molted and all died. To test whether these larvae could resume development upon regaining the ability to produce miRNAs, we shifted mirtron-rescued *pash-1(ts)* L1 larvae back to the permissive temperature. This enabled a quarter of the larvae to become fertile adults, suggesting that L1 larvae rescued by mirt-35 and mirt-51 during embryogenesis are competent to complete development (Figure S3H) and that additional miRNAs are required for larval progression (in addition to *lin-4* and *let-7*, whose loss causes specific defects distinct from those observed here) [36, 37].

**Figure 4:**
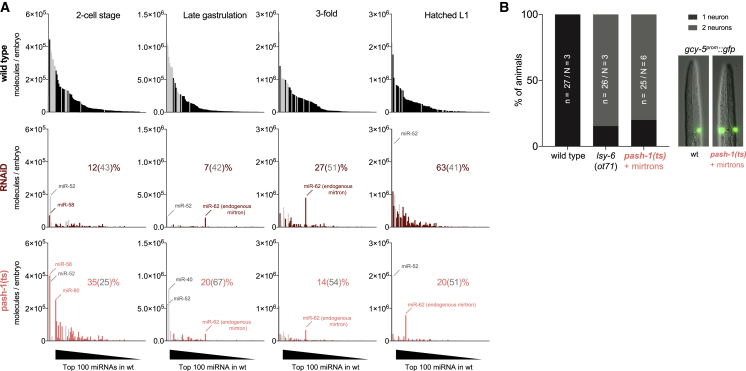
Mirtron-Rescued Embryos Lack Canonical miRNAs at the Molecular and Functional Level (A) Absolute miRNA abundance in WT, RNAiD, and *pash-1(ts)* animals under restrictive conditions, at the indicated time points, measured by spike-in normalized small RNA-seq as in Figure 1B. Shown are the top 100 miRNAs, ranked by abundance in WT samples. Remaining miRNAs of the miR-35 and -51 families are in gray. For comparison, data for 2-cell and gastrulation time points are replotted from Figures 1B and S1G. The percentage of miRNA molecules remaining relative to WT is indicated, with the relative contribution of mir-35 and mir-51 families combined noted in brackets. For quantification of miRNAs grouped by families, see Figure S4A. (B) Functional readout for the function of the late embryonic miRNA *lsy-6*. In WT animals, only the right ASE neuron expresses *gcy-5:gfp*, but absence of functional *lsy-6* results in activation of the reporter in both the left and right neurons.

The ability to produce rescued larvae enabled us to assess the level of miRNA depletion beyond the gastrulation time point. We performed quantitative small RNA sequencing in 3-fold embryos (8 h post-2-cell) and in hatched L1 larvae, in addition to the 2-cell and late gastrulation time points (Figures 4A and S4A). Note that the depletion data plotted in Figure 1B are part of this full time course obtained from mirtron-expressing embryos, as we determined that mirtron expression did not affect the levels of other miRNAs under Microprocessor depletion conditions (Figure S4B). Mirtron-rescued 3-fold embryos and L1 larvae of the *pash-1(ts)* strain were strongly depleted of miRNAs (14% and 20% of miRNA molecules remained compared to wild type [WT], of which 54% and 51% were miR-35/51^fam^ members, respectively). Notably, two miRNAs persisted, the miR-51^fam^ member miR-52, which is partially resistant to Microprocessor ablation, and miR-62, an endogenous mirtron without obvious function [25]. RNAiD treatment, which was very efficient for depletion of maternal and zygotic miRNAs up to the end of gastrulation, still caused significant miRNA depletion in 3-fold embryos (27% remaining, of which 51% correspond to miR-35/51^fam^ members) but was less effective for miRNA depletion in L1 larvae, likely because auxin levels in late embryos become insufficient to trigger efficient degradation. From the combination of the RNAiD and the *pash-1(ts)* experiments, we conclude that miR-35^fam^ and miR-51^fam^ are sufficient for embryonic development in the absence of Drosha and Pasha/DGCR8 activities.

We also confirmed that mirtron-rescued larvae were functionally depleted of miRNAs that act later in cell-type-specific manners. For instance, *lsy-6* is a miRNA necessary to lateralize the ASE pair of neurons [38]. Mirtron-rescued *pash-1(ts)* larvae behaved identically to larvae bearing a genomic deletion of *lsy-6* in their inability to properly specify the ASE lateral asymmetry (Figure 4B). Hence, these larvae, which lack practically all miRNAs except for miR-35/51^fam^, also provide an opportunity to explore the global contribution of such later-acting, cell-type-specific miRNAs.

## Discussion

We showed that Microprocessor-depleted *C. elegans* embryos arrest shortly after gastrulation, failing to undergo morphogenesis or to form organs. This arrest phenotype is earlier than in Dicer-deficient zebrafish, which manage to form most organs, albeit imperfectly [17]. The early arrest is reminiscent of Pasha/DGCR8 mutant mouse embryos, which die during gastrulation [5]. Two conserved miRNAs, one member of each miR-35^fam^ and miR-51^fam^, are sufficient to overcome this arrest in *C. elegans*.

Both Drosha and Pasha/DGCR8 bind a variety of non-miRNA substrates [9, 10, 11] and perform regulatory functions through cleavage or binding of mRNAs [12, 13, 14, 15]. However, the biological significance of these functions remained unclear. As embryonic lethality caused by absence of Pasha and Drosha is rescued by addition of miR-35 and miR-51, we conclude that neither protein carries out essential miRNA-independent functions during early *C. elegans* embryogenesis.

The miR-35^fam^ and miR-51^fam^ are broadly conserved: miR-35^fam^ is found across Protostomia and miR-51^fam^ is part of the miR-100^fam^, one of the most conserved families across all animals [27, 39]. Yet the functions of these two miRNA families remain poorly understood. In *C. elegans*, miR-35^fam^ has been implicated in fecundity, sex determination, and apoptosis [23, 40, 41, 42, 43]. However, none of these functions explain the fully penetrant embryonic lethality. The role of miR-51^fam^ remains less explored; part of the defects observed in *C. elegans* embryos lacking this family are related to the fat cadherin ortholog, CDH-3 [27]. However, deregulation of CDH-3 does not explain the embryonic lethality in the absence of miR-51^fam^.

Zebrafish miR-430 is expressed in the zygote, where it plays a role in maternal mRNA clearance [18]. In contrast, miR-35^fam^ and miR-51^fam^ are present in the germline, and at least for miR-35^fam^, there is evidence that it is active in the maternal germline [42, 43]. Moreover, the levels of the predicted targets of the miR-35^fam^ and miR-51^fam^ tend to stay constant during embryogenesis or even increase (Figure S4C) [44]. These observations strongly suggest that miR-35^fam^ and miR-51^fam^ do not function in maternal mRNA clearance. These two miRNA families thus play a yet unidentified, likely conserved function in animal embryogenesis.

We propose that the finding that two miRNAs are sufficient for early embryonic patterning in *C. elegans*, together with the predominance of miR-430 in zebrafish embryogenesis [17], reflects a more general principle of miRNA function in animal development, namely that only few miRNAs have acquired functions in the early stages of embryogenesis. Notably, these miRNAs with early, essential functions are present as families with multiple members that share the same seed sequence and are expressed broadly and abundantly. Consistently, in the mouse, the only miRNAs known so far to play a role in early patterning are the miR-290 and miR-302 clusters [45]; deletion of both clusters simultaneously causes arrest around E6.5, similar to the loss of DGCR8 [5].

Whereas few miRNAs act at early stages of embryogenesis, we propose that the majority of miRNAs in *C. elegans*, but also in other animals, play roles in the development and physiology of specialized cells. Most miRNAs in *C. elegans* are expressed with high cell type specificity at later stages of embryogenesis and/or in differentiated cells [16, 28, 29]. Although not strictly required for survival under laboratory conditions, such cell-specific miRNAs contribute to the acquisition of the phenotypic diversity that is critical for the animal’s evolutionary success in complex natural environments (e.g., by enabling development and function of diverse sensory neurons) [38, 46]. Similarly, many miRNAs in other animals are expressed with high cell type specificity [47, 48] and, based on a comprehensive survey in the mouse [1], seem to function in cell- or tissue-specific development or physiology. Many of these mouse miRNAs are also essential for viability, although lethality in these cases occurs late in development in correctly patterned embryos that fail to generate specific cell types, e.g., miR-1-deficient mice die shortly after birth due to cardiac muscle defects [49]. In contrast in *C. elegans*, most miRNAs are not required for viability in the lab, even at later stages [25]. This distinction between worms and mice likely reflects a difference in the tolerance of each organism to defects in the production of differentiated cells rather than generally distinct roles of miRNAs (e.g., miR-1-deficient *C. elegans* also show a range of molecular and cellular defects in muscles, but the animals are still fully viable in the lab) [50, 51, 52].

Our work supports the distinction of two classes of miRNAs that contribute to the production of a multicellular organism in different ways: few miRNAs act in the early stages of development, whereas the majority of miRNAs play more specialized roles at later stages [16]. This classification may provide a more general framework for understanding the contributions of miRNAs to animal development and evolution.

## STAR★Methods

### Key Resources Table

**Table:** 

REAGENT or RESOURCE	SOURCE	IDENTIFIER
**Antibodies**		

Mouse monoclonal anti-myc (clone 4A6)	Merck Millipore	Cat# 05-724; RRID: AB_11211891
Rabbit polyclonal anti-gamma-tubulin	Abcam	Cat# ab50721; RRID: AB_880628)
Anti-mouse IgG secondary HRP-linked	Cell Signaling Technologies	Cat# 7076; RRID: AB_330924)
Anti-rabbit IgG secondary HRP-linked	Cell Signaling Technologies	Cat# 7074; RRID: AB_2099233)

**Bacterial and Virus Strains**		

*E. coli* OP50 standard *C. elegans* food source	CGC	N/A
*E. coli* HT115 RNAi bacteria (targeting pash-1)	ORFeome version 1.1	T22A3.5 (ORF-ID)

**Chemicals, Peptides, and Recombinant Proteins**		

3-Indolacetic acid (Auxin)	Sigma-Aldrich	CAS I2886
Cas-9 from *Staphylococcus aureus*	In-house	N/A

**Critical Commercial Assays**		

TRIzol Reagent	Invitrogen	CAS 15596026
SuperScript III	Invitrogen	CAS 18080044
GoTAQ qPCR Mastermix	Promega	CAS A6001
Alt-R CRISPR-Cas9 tracr/crRNA system for genome editing (insertion of AID-tags)	Integrated DNA technologies	N/A

**Deposited Data**		

Small RNAseq data deposition with NCBI GEO	This paper	GEO: GSE153233

**Experimental Models: Organisms/Strains**		

*C. elegans* strains used/generated: see Table S1	CGC / this paper	N/A

**Oligonucleotides**		

qPCR-primer: pash-1-F: 5′-GCCTTCGAGAAAACG GGGAA-3′	This paper	N/A
qPCR-primer: pash-1-R: 5′-TGGCTCCCATTTCGG AGATT-3′	This paper	N/A
qPCR-primer: drsh-1-F: 5′-TGAGCTGGCTTTGGCT AATCT-3′	This paper	N/A
qPCR-primer: drsh-1-R: 5′-ACCCCGTAATTAGTCG CTGG-3′	This paper	N/A
qPCR-primer: cdc-42-F: 5′-TGGGTGCCTGAAATT TCGC-3′	This paper	N/A
qPCR-primer: cdc-42-R: 5′-CTTCTCCTGTTGTGG TGGG-3′	This paper	NA
ALT-R crRNA drsh-1 N terminus: 5′- TTAGATTTTCATTT AGATGT-3′	This paper	N/A
ALT-R crRNA drsh-1 C terminus: 5′-GATACCAGCGACT AATTACG-3′	This paper	N/A
ALT-R crRNA pash-1 C terminus: 5′-AGGTGAATATACT ATTTGTG-3′	This paper	N/A

**Recombinant DNA**		

Plasmid pPD75_95 (base vector for mirtrons)	Fire Lab / Addgene	CAS 1494
Expression vectors for mirtron-35 and mirtron-51 (see STAR Methods or Figure S2 for details)	This paper	N/A

**Software and Algorithms**		

RNAfold Two-State-Folding algorithm	[53]	http://unafold.rna.albany.edu/
miRNA analysis pipeline for sRNaseq data	[28]	https://doi.org/10.1038/nmeth.4610
Nextflow pipeline	[54]	https://www.nextflow.io/about-us.html
Nextflow pipeline modifications	This paper	https://github.com/lengfei5/smallRNA_nf/tree/master/dev_sRBC
Scripts for analysis of sRNaseq data presented here	This paper	https://github.com/lengfei5/smallRNA_analysis_philipp/tree/master/scripts

### Resource Availability

#### Lead Contact

Further information and requests for resources and reagents should be directed to and will be fulfilled by the Lead Contact, Luisa Cochella (cochella@imp.ac.at).

#### Materials Availability

Key strains will be made available at the Caenorhabditis Genetics Center (University of Minnesota), other strains and plasmids are available upon request.

#### Data and Code Availability

Sequencing data are deposited under the GEO accession number GEO: GSE153233. Small RNA reads were mapped and processed as in [28]. Modifications of the Nextflow pipeline for can be found under: https://github.com/lengfei5/smallRNA_nf/tree/master/dev_sRBC. Other scripts can be found at: https://github.com/lengfei5/smallRNA_analysis_philipp/tree/master/scripts

### Experimental Model and Subject Details

*Caenorhabditis elegans* was kept under standard conditions on NGM plates seeded with *Escherichia coli* OP50 at 20°C as previously described [55], unless indicated otherwise. The Bristol strain N2 was used as a wild-type control. MiR-35 family mutant embryos were derived from self-fertilized, homozygous mutant mothers (nDf49, nDf50) that were maternally rescued. For analysis of the earliest miR-51 family mutant phenotypes, we sought to reduce the maternal load of miR-51 family miRNAs (as embryos can only be obtained from mothers expressing at least one mir-51 family member). To this end, mutant embryos were derived from mothers expressing miR-54-56 as well as an early embryonic mir-35^prom^::GFP reporter from an extrachromosomal array, while all genomic copies of miR-51 family miRNAs were deleted (nDf58, nDf67, n4100). These arrays tend to be lowly expressed or silenced in the *C. elegans* germline and are only segregated to a fraction of the offspring. Therefore, miR-51 family mutant embryos obtained from these mothers upon self-fertilization were selected for lack of the rescuing array by absence of mir-35^prom^::GFP expression, and subsequently analyzed. A full list of strains used and generated in this study is provided in Table S1.

### Method Details

#### Mirtron design and expression

Production of a miRNA of interest via the mirtron pathway involves the challenge of designing an intron that resembles a pre-miRNA hairpin and simultaneously allows for efficient splicing. To generate mirtron-versions of miR-35 or miR-51, the 6 nucleotides at the 3′ end of the respective pre-miRNA-hairpin were modified to create a 3′ splice site and match the consensus sequence of 12 annotated endogenous *C. elegans* mirtrons (Figure S2A) [56]. Because intron borders correspond to the ends of mature miRNAs, the 5′ arm of mirtrons invariably starts with a G, which negatively impacts loading into Argonaute [57]. Thus, mature mirtrons are preferably incorporated into the 3′ arm. As interaction of miRNAs with cognate target mRNAs is largely determined by the seed region (nts 2-8), minor alterations in the 3′ end of a miRNA are expected to have little impact on its target repertoire. As branch point motifs are rather permissive in *C. elegans*, virtually any A located in vicinity to a 3′ splice site allows for efficient lariat formation [58], we thus made no additional changes to account for the branch point. Upon introduction of a functional 3′ splice site into the 3p-arm of the pre-miRNA, compensatory changes were introduced in the 5p arm to retain hairpin secondary structure. This was guided by secondary structure predictions using the RNAfold Two-State-Folding algorithm for RNA at 20°C [53], yielding a mirtron-hairpin with a secondary structure highly similar to its endogenous pre-miRNA-hairpin equivalent (Figure S2C). The resulting sequence was used to replace the middle intron of GFP in the commonly employed *C. elegans* vector pPD75.95 (Fire lab). To recapitulate the spatio-temporal expression pattern of endogenous miRNAs, the promoter sequence of the respective endogenous miRNA was used to drive mirtron expression (2.0 kb upstream of pre-mir-35 or 2.5 kb upstream of pre-mir-52, respectively). Conveniently, GFP-fluorescence serves as an internal control for correct splicing of the inserted mirtron. Functionality of mirtrons was assayed by testing their ability to rescue an otherwise lethal deletion of the respective miRNA family. For the design of mirtron-35_seed mutant_, three base pairs in the seed region of mirtron-35 were swapped in the pre-mirtron stem-loop, preserving integrity and thermodynamic features of the hairpin and miRNA-duplex to ensure efficient processing and loading of the mirtron-variant.

#### Genome engineering

To generate AID-tagged copies of *pash-1* and *drsh-1,* animals were subjected to Cas-9 mediated genome engineering via RNP-microinjection following the Co-CRISPR approach described by [59], with modifications from [60]. Briefly, the Alt-R CRISPR/Cas9 system (Integrated DNA Technologies) was employed and animals were injected with a mixture containing 300mM KCl, 20mM HEPES, 4μg/μl recombinant Cas9 (from *S. aureus,* purified in-house), 500ng/μl TracerRNA, 100ng/μl crRNA targeting the Co-CRISPR marker gene dpy-10, and 15ng/μl dpy-10- > rol-6 repair template oligo. Additionally, 100 ng/μl crRNA against the locus of interest as well as 100-200ng/μl dsDNA repair template encoding the desired modification with around 50bp flanking homology arms were added to the mix. Note that for efficient degradation of DRSH-1 we needed to insert AID degrons at the N- and C-termini simultaneously as either degron alone was insufficient for depletion. Guide RNAs used and sequences inserted (in bold) were as follows:

*drsh-1* – N-terminal AID-tag – guide sequence used = 5′- TTAGATTTTCATTTAGATGT-3′ – locus post-edit:
TAGTTAACGTTTTCATCTGAAATTGTAGACAGATTTAGATTTTCATTTAG**ATGGAACAGAAACTCATCTCTGAAGAGGATCTG*ATGCCTAAAGATCCAGCCAAACCTCCGGCCAAGGCACAAGTTGTGGGATGGCCACCGGTGAGATCATACCGGAAGAACGTGATGGTTTCCTGCCAAAAATCAAGCGGTGGCCCGGAGGCGGCGGCGTTCGTGAAG*GACTACAAAGACCATGACGGTGATTATAAAGATCATGACATCGACTACAAGGATGACGATGACAAGGGAGGAAGCGGAGGAGGAAGCGGTGGAGGAAGCGGAGGAGGAAGCGGA**ATGTCGGACGAAAAGATTTCAATGACGCTTAACTTCCCGAAACACAAGCG
*drsh-1* – C-terminal AID-tag - guide sequence used = 5′-GATACCAGCGACTAATTACG-3′ – locus post-edit:
TCAAGTGGTTTCAGAACATGCGCCGTCGTCTTGAACAAGATACCAGCGACGGAGGCAGCGGTGGTGGAAGCGGCGGGGGAAGCGGCGGTGGAAGCGGTGACTACAAAGACCATGACGGTGATTATAAAGATCATGACATCGACTACAAGGATGACGATGACAAG*ATGCCTAAAGATCCAGCCAAACCTCCGGCCAAGGCACAAGTTGTGGGATGGCCACCGGTGAGATCATACCGGAAGAACGT****GATGGTTTCCTGCCAAAAATCAAGCGGTGGCCCGGAGGCGGCGGCGTTCGTGAAG*GAACAGAAACTCATCTCTGAAGAGGATCTGTAG**TTACGGGGTTATAATTATACTATGTCTGTTTGAATGTGATTCGGTTC
*pash-1* – C-terminal AID-tag – guide sequence used = 5′-AGGTGAATATACTATTTGTG-3′ – locus post-edit:
GGGGAAGACATGACGATTCATCATCCCCATCACATCAGAAACCACACAAA**GGAGGAAGCGGAGGAGGAAGCGGAGACTACAAAGACCATGACGGTGATTATAAAGATCATGACATCGACTACAAGGATGACGATGACAAG*ATGCCTAAAGATCCAGCCAAACCTCCGGCCAAGGCACAAGTTGTGGGATGGCCACCGGTGAGATCATACCGGAAGAACGTGATGGTTTCCTGCCAAAAATCAAGCGGTGGCCCGGAGGCGGCGGCGTTCGTGAAG*GAACAGAAACTCATCTCTGAAGAGGATCTGTAG**TATATTCACCTCATATGTTTGTTGTTTTGTTGGTAGTTTTAATTTTT
Inserted sequences contained the TIR-1 recognition peptide (italics), a FLAG (GACTACAAAGACCATGACGGTGATTATAAAGATCATGACATCGACTACAAGGATGACGATGACAAG) and a MYC (GAACAGAAACTCATCTCTGAAGAGGATCTG) tag, as well as GGSG linkers in between.

#### Pash-1(ts) experiments

Animals bearing the *pash-1(ts)* allele *mj100* were kept constantly at the permissive temperature of 16°C. *pash-1(ts)* animals expressing the mirtron-51 transgene were selected every few generations for high levels of the co-expression marker *elt-2p::dsRed*, as this transgene was prone to undergo silencing. For experiments, L4 stage larvae were transferred to a fresh plate and shifted to 25°C approximately 16h later. In order to achieve maximal depletion of maternal miRNAs, mothers were kept at the restrictive temperature an additional 7h before harvesting early embryos (1-4 cell stage). Extending the time at the restrictive temperature was prohibited by the rapidly deteriorating health of gravid *pash-1(ts)* adults expressing mirtrons at 25°C. For backshift-experiments with mirtron-rescued *pash-1(ts)* animals, hatched L1s that developed under restrictive conditions were transferred to a fresh plate within 1h after hatching, allowed to develop at 16°C, and scored for their ability to reach adulthood within 7 days.

#### RNAiD experiments

Animals were kept constantly on standard NGM plates seeded with *E. coli* OP50 at 20°C. For combined AID and RNAi treatment, NGM plates were supplemented with 1mM Carbenicillin, 1mM IPTG and 4mM Auxin (3-Indoleacetic acid, Sigma #I2886). RNAiD plates were seeded with *E. coli* HT115 expressing dsRNA which elicits an RNAi response against *pash-1* mRNA (clone from the ORFeome-RNAi v1.1 (Vidal) library, ORF-ID T22A3.5). To ensure efficient mRNA knockdown, RNAi bacteria were grown freshly for every experiment in liquid LB culture, the medium was brought to 1mM IPTG around 1h before pelleting bacteria, and plates were seeded with a 20X concentrate (as Auxin inhibits bacterial growth). For experiments, L4s were transferred to RNAiD plates, embryos were harvested 24h later, and assayed as described below. While the AID-system rapidly depletes proteins within minutes to hours [20], extending the Auxin treatment to 24h was required to ensure near-complete elimination of maternal miRNAs. Surprisingly, overall health of mirtron-expressing gravid adults upon RNAiD treatment was significantly less adversely affected compared to *pash-1(ts)* animals, eventually owed to the temperature difference and/or deleterious side-effects of PASH-1(TS) at 25°C. Efficiency of RNAi-mediated *pash-1* mRNA knockdown was assessed via RT-qPCR. Briefly, 5-10 embryos in the 2-cell stage were transferred to about 1 μl of Lysis Buffer (5mM Tris-HCl pH 8.0, 0.25 mM EDTA and 1 mg/mL Proteinase K, 0.5% Triton X-100, 0.5% Tween20) using a thin glass needle. These samples were subjected to 10 min digestion at 65°C before heat inactivation of proteinase K for 1 min at 85°C. Next, crude lysates were reverse transcribed before performing qPCR using the GoTAQ qPCR Mastermix (Promega) according to the manufacturer’s instructions. Relative expression was calculated according to the ΔΔCq-method using *cdc-42* as a reference gene, the following primer sequences were employed:

*pash-1*-F: 5′-GCCTTCGAGAAAACGGGGAA-3′; *pash-1*-R: 5′-TGGCTCCCATTTCGGAGATT-3′;
*drsh-1*-F: 5′-TGAGCTGGCTTTGGCTAATCT-3′; *drsh-1*-R: 5′-ACCCCGTAATTAGTCGCTGG-3′
*cdc-42*-F: 5′-TGGGTGCCTGAAATTTCGC-3′; *cdc-42*-R: 5′-CTTCTCCTGTTGTGGTGGG-3′

#### Western blotting

To assess the extent of protein degradation of PASH-1:AID:MYC and DRSH-1:AID:MYC upon Auxin treatment, L4s were transferred to Auxin plates prepared as described above. 24h later, gravid adults were collected in Lämmli-buffer containing 2.5% β-Mercaptoethanol (v/v), and subjected to multiple cycles of snap-freezing followed by boiling until embryos were disrupted. Proteins were separated via SDS-PAGE and transferred onto a Nitrocellulose membrane. As primary antibodies either monoclonal mouse anti-myc (clone 46A, Merck Millipore, catalog number 05-724, diluted 1:2000) or a polyclonal rabbit anti-gamma tubulin (Abcam, catalog number ab50721, diluted 1:1,000) were used. As secondary antibody, anti-mouse IgG HRP-linked Antibody (Cell Signaling Technology, #7076, diluted 1:2,000) or anti-rabbit IgG HRP-linked Antibody (Cell Signaling Technology, #7074, dilution of 1:2,000) was applied followed by visualization using ECL reagent (Thermo Scientific).

#### Hatching assays

Embryos were obtained from day 1 gravid adults reared as described above, by slicing mothers in a drop of M9 buffer on a microscopic slide. 2-cell embryos of normal size were transferred to standard NGM plates or RNAiD plates and allowed to develop at the respective temperatures. Hatched animals were collected for subsequent analysis (backshift experiments, microscopy, or small RNA sequencing) from plates within 1h after hatching. The final hatching rate was assessed > 24h after embryo collection by scoring the fraction of animals that successfully escaped the eggshell. To obtain samples at specific developmental stages for small RNA sequencing, 2-cell embryos were harvested via slicing, collected or allowed to develop on NGM plates before being selected manually by stage at given time points (gastrulation: 4-5h after 2-cell stage, 3-fold = 8-9 hours after 3-cell stage).

#### Microscopy

For phenotypic analysis of embryonic arrest or hatched L1 larvae, animals were mounted on a thin agar-pad on a microscopic slide sealed with a coverslip. Images were recorded at 400x magnification using an AxioImager Z2 (Zeiss) equipped with DIC and fluorescence optics, and analyzed via ImageJ. Arrest phenotypes were scored as follows: cell mass = embryo fails to initiate bean stage; morphogenesis = embryo begins to elongate but fails to reach 2-fold stage; elongation = embryo completes 2-fold stage but arrests before hatching. Body measurements are based on DIC micrographs. Body length was measured as the length of a segmented path running through the mid body axis from head to tail. Body width was assessed as the arithmetic mean of 3 independent width measurements in the anterior, middle, and posterior part of each animal. Pharynx length was determined by the length of a segmented path running through the pharynx middle axis in animals expressing *myo-2*^*prom*^*::mCherry*. Mean and range are plotted for each genotype; unpaired t test was used for statistical comparison.

#### Small RNA sequencing

To profile miRNA levels, a modified version of the small RNA sequencing protocol described in [28] was performed. Samples containing each between 25 and 100 embryos (yielding about 5 to 20 ng total RNA) were collected at indicated stages and snap-frozen in liquid nitrogen. A series of eight RNA spike-In oligos spanning a 500-fold range of concentrations [61] were added on a per embryo basis, and total RNA was extracted using TRIzol Reagent (Invitrogen) according to the recommendations by the manufacturer for cell samples. To ensure complete disruption of embryos, samples were snap-frozen and thawed multiple times before proceeding with the extraction. After total RNA purification samples were treated as described in [28], with two important changes: A) amounts of 3′ linker and 5′ linker were reduced to a final concentration of 500 nM to reduce undesired amplification products. B) barcodes and random nucleotides serving as unique molecular identifiers were introduced into the 3′ linker to circumvent low-input related contamination and over-amplification issues, resulting in ligation products of the following sequence:

(XXXXX = barcode): 5′-ACACUCUUUCCCUACACGACGCUCUUCCGAUCUNNNN-(sRNA)_18-30nt_-NNNNNNXXXXXAGATCGGAAGAGCAC-ACGTCT/3ddC/-3′. After sufficient PCR-amplification as observed by SYBR green derived qPCR signal (requiring around 16-20 cycles), libraries were size-selected on an agarose gel to remove adaptor dimers and sequenced on a HiSeqV4 platform (Illumina). Small RNA reads were mapped and processed as in [28]. Modifications include addition of 3′ adaptor-barcode demultiplexing, which was added after trimming adaptor sequence in the Nextflow-based pipeline [54]. The modified Nextflow pipeline can be found at: https://github.com/lengfei5/smallRNA_nf/tree/master/dev_sRBC. To accurately quantify sRNAs, both mapped sequences and associated random nucleotides (functioning as UMIs) within the raw reads were quantified for miRNAs and piRNAs as well as spike-ins. To alleviate PCR amplification bias, UMI counts were used and normalized to spike-in as described in [61], allowing for absolute quantification of miRNA content per embryo even under conditions in which most miRNAs are depleted. In addition, as piRNAs are not affected across conditions and can thus be considered an internal control, data was normalized in parallel using total number of piRNA reads, yielding results highly similar to the ones obtained by spike-in normalization and providing additional confidence in the quantitative nature of the data. Scripts for this analysis can be found at: https://github.com/lengfei5/smallRNA_analysis_philipp/tree/master/scripts. Every round of library preparation included one sample of a comparable amount of *Arabidopsis thaliana* total RNA to assess the extent of potential contamination. The average counts for reads mapping to *C. elegans* miRNAs in contamination control samples (∼1% of the wild-type miRNA content) across four independent library preparations were subtracted from all samples as a background correction. For background-corrected, spike-in normalized, miRNA counts in molecules per embryo, see Table S2. Raw data from sRNA-seq experiments has been deposited in the NCBI-GEO database under the accession number GEO: GSE153233.

### Quantification and Statistical Analysis

Plots were created using Prism, statistical analysis was also performed in Prism as described in the figure legends. For sRNaseq data statistical analysis was performed using DESeq2 [62]. Figures were prepared with Adobe Illustrator CS6.

## Supplemental Information

Document S1. Figures S1–S4 and Table S1

Table S2. Processed Small RNA Sequencing Data, Related to STAR Methods Output of the pipeline used to extract miRNA reads from sRNaseq raw data. miRNA reads are normalized to spike-ins and denoted in molecules / embryo.

Document S2. Article plus Supplemental Information

Video Abstract
