# Supplementary material for: Two MicroRNAs Are Sufficient for Embryonic Patterning in C. elegans
Source: Curr Biol. 2020 Dec 21;30(24):5058–5065.e5. doi: 10.1016/j.cub.2020.09.066 (PMC7758728; doi:10.1016/j.cub.2020.09.066)
Supplement: Document S1. Figures S1–S4 and Table S1 [file mmc1.pdf]

Current Biology, Volume *30*

Supplemental Information

Two MicroRNAs Are Sufficient for Embryonic Patterning in *C. elegans*

Philipp J. Dexheimer, Jingkui Wang, and Luisa Cochella

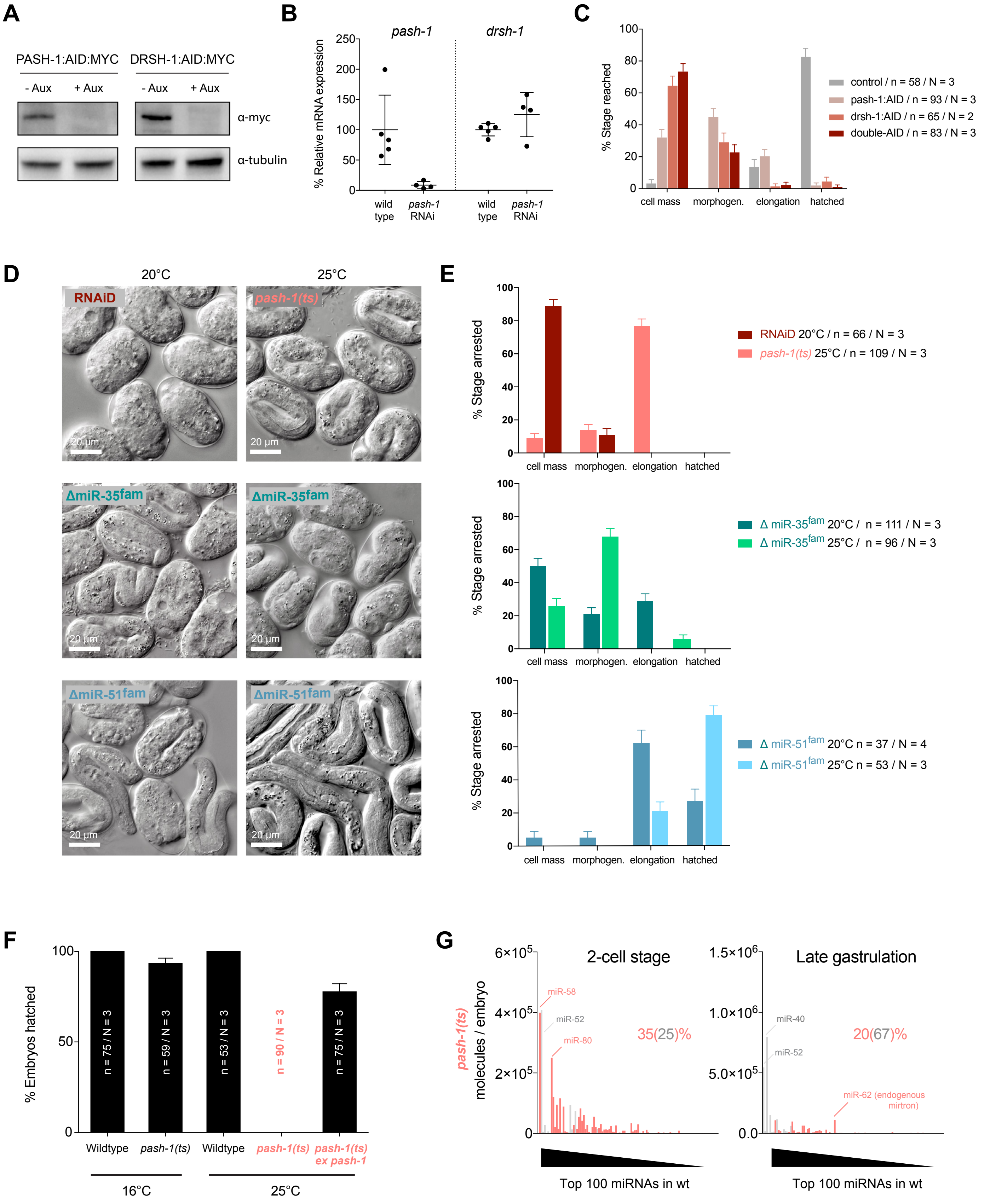

Figure S1. miRNAs are essential for morphogenesis and organogenesis. Related to Figure 1. A) Western Blot against endogenous, AID-Myc-tagged PASH-1 and DRSH-1 in gravid adults, 24h post Auxin treatment. **B**) mRNA levels measured by RT-qPCR in 2-cell embryos, 24h post pash-1 RNAi treatment compared to wild type under standard conditions. **C**) Quantification of the arrest phenotype of various strains bearing AID-tagged PASH-1 and/or DRSH-1 and expressing the TIR1 ubiquitin ligase, or TIR1 only as control, upon Auxin treatment. **D, E**) Images and quantification of arrest phenotype of RNAiD, *pash-1(ts)*, miR-35^{fam}, and miR-51^{fam} mutant embryos at 20°C and 25°C. **F**) Hatching assay of embryos bearing the pash-1(ts) temperature-sensitive allele mj100. Penetrant embryonic lethality at the restrictive temperature 25°C is rescued by expression of wild-type pash-1 from an extra-chromosomal array. **G**) miRNA-profile of pash-1(ts) embryos at the 2-cell stage and at the end of gastrulation as determined by small RNA sequencing using spike-in oligos for quantitative measure of absolute miRNA levels across samples, plotted as in Figure 1B. Note that the samples analyzed here are the same as in Figures S2G, 4A, S4A.

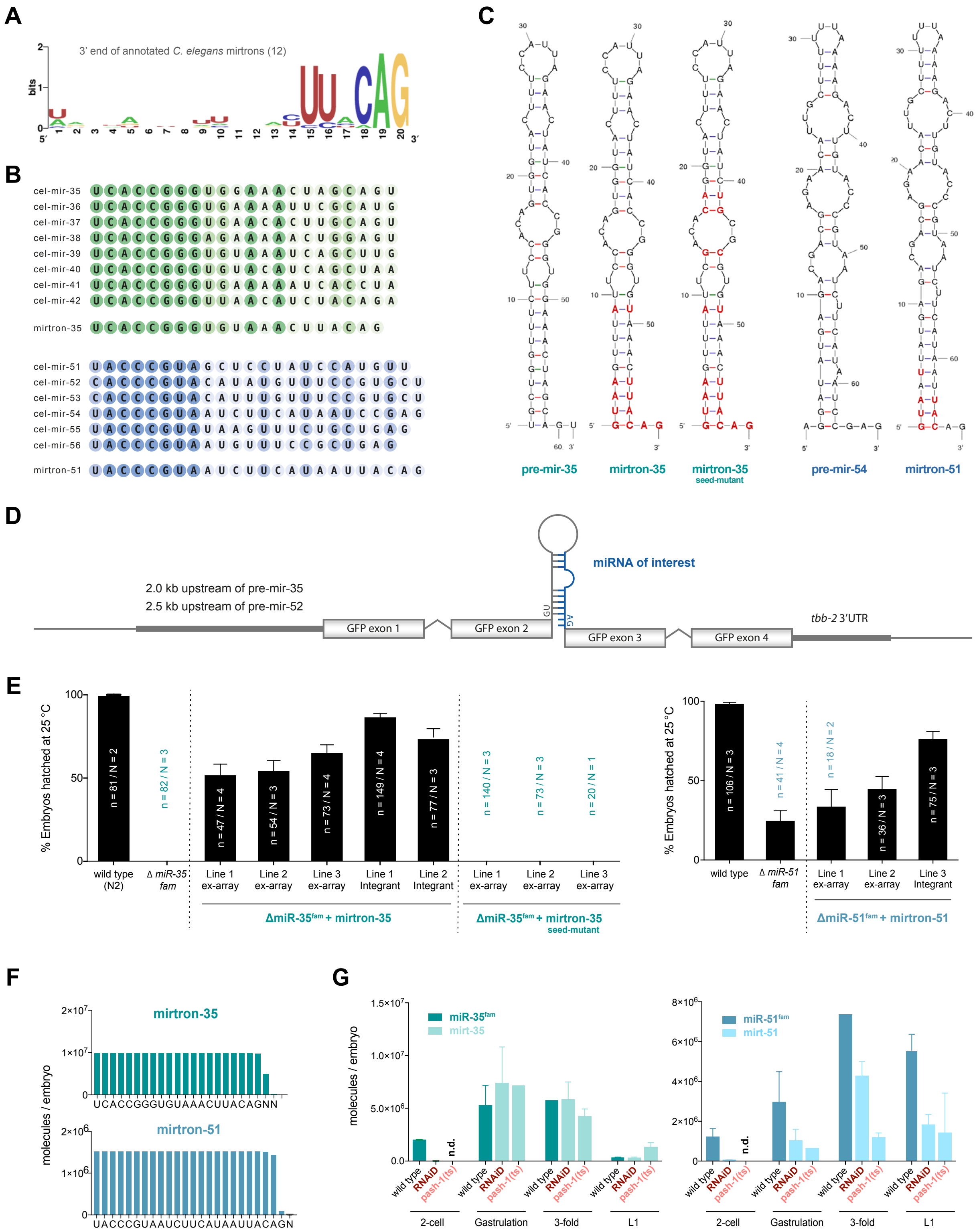

Figure S2. Mirtron design and expression. Related to Figure 2. A) Consensus sequence at the 3′ end of all 12 annotated *C. elegans* mirtrons (S1). **B**) Sequence of miR-35 and miR-51 family miRNAs and the corresponding designed mirtrons. **C**) Pre-miRNA hairpin structure for endogenous miRNA and mirtron counterpart as predicted by the UnaFold TwoState folding algorithm for RNA at 20ºC (S2); the basis for the design of mirtron-51 was the family member miR-54 as it required relatively few changes to be turned into a mirtron. Residues that have been changed in the mirtron hairpins are highlighted in red. **D**) Schematic design of mirtron-expressing transgenes. The endogenous promoter sequence upstream of *pre-mir-35* (2.0 kb) or *pre-mir-52* (2.5 kb) drives expression of a GFP transcript bearing the mirtron in its middle intron, followed by an inert *tbb-2* (tubulin) 3′ UTR. **E**) Hatching rates of embryos bearing deletions for the miR-35 family (*nDf49, nDf50*), or the miR-51 family (*nDf58, nDf67, n4100*), respectively. Shown are multiple independent transgenic lines expressing the respective mirtron from an extra-chromosomal array or the integrated transgenes that were used for subsequent experiments. **F**) Mirtron-read abundance per nucleotide derived from sRNA-seq data from mirtron-expressing RNAiD embryos at gastrulation (mirtron read-profiles were highly similar for other time points). Mirtron-35 had an untemplated single-nucleotide addition in 25-45% of the reads, which was a U in >90% of cases, consistent with observations in *Drosophila* (S3, S4). Mirtron-51 was trimmed by one nucleotide in 90-95% of cases. **G**) Comparison of the sum of the abundances of all endogenous miRNA family members in wild-type embryos (dark shades) and the respective mirtron abundance in Microprocessor depleted animals (light shades) throughout embryogenesis, derived from quantitative sRNA-seq analysis. Mirtrons are not detected at the 2-cell stage, consistent with commonly observed silencing of multicopy transgenes in the *C. elegans* germline. Thus, germline-related phenotypes such as the loss in fertility are not rescued by the mirtron transgenes.

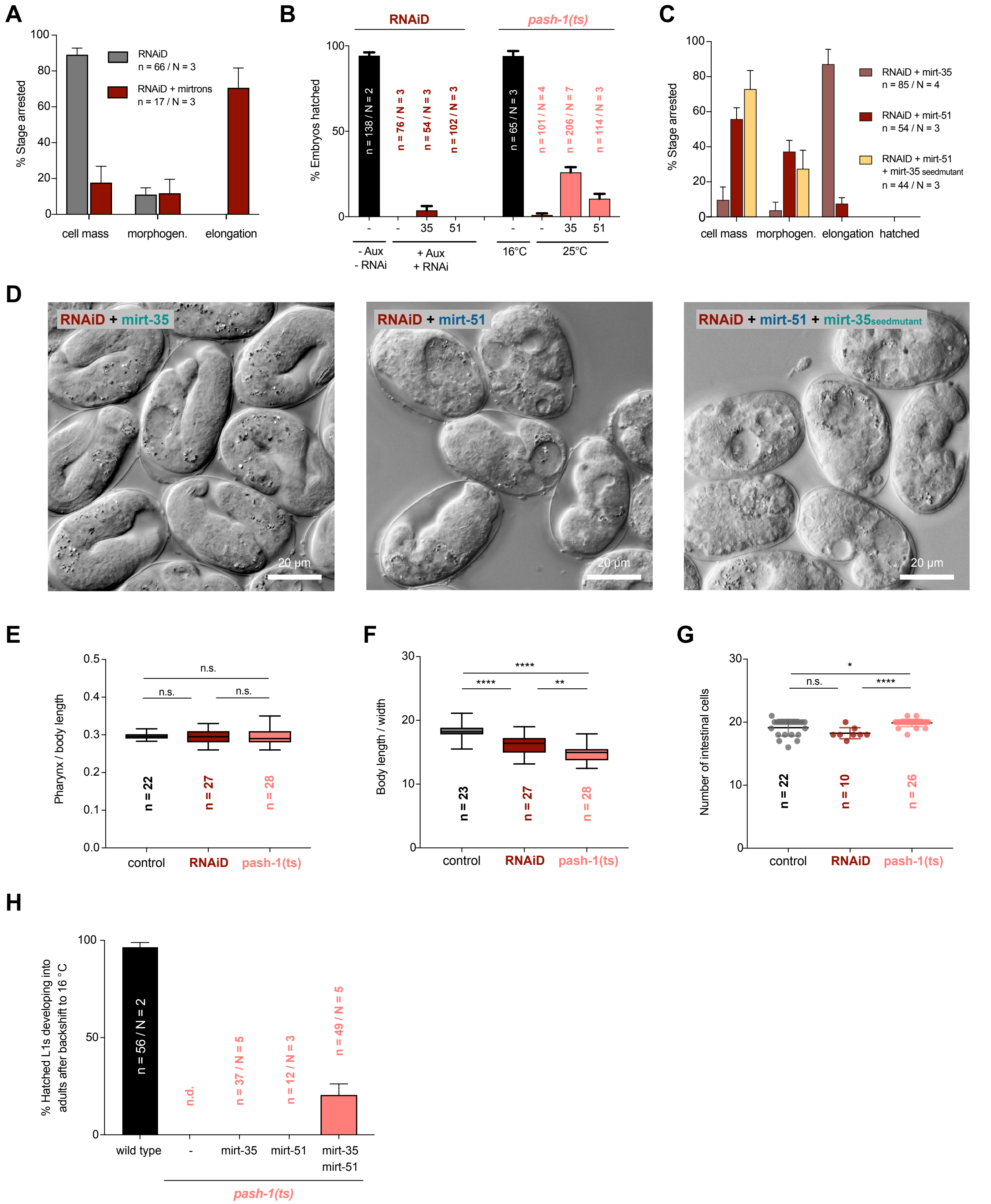

Figure S3. Two miRNAs are sufficient for embryonic patterning in the absence of Drosha and Pasha. Related to Figure 3. **A**) Stage of arrest in the ~50% of RNAiD embryos with mirtron-35 and -51 that fail to hatch, compared to embryos without mirtrons. For comparison data of RNAiD embyros is replotted from Figure S1E. **B**) Proportion of hatching of Microprocessor-deficient embryos expressing either mirtron-35 or mirtron-51 individually. **C**) Stage of arrest of RNAiD embryos expressing either mirtron individually, or mirtron-51 and the seed-mutant version of mirtron-35. For the seed-mutant mirt-35, three independent lines behaved identically of which one is shown. **D**) Representative images of animals scored in C. **E-G**) Body measurements of mirtron-rescued RNAiD or *pash-1(ts)* animals compared to wild type under standard conditions. Data is derived from micrographs using ImageJ. Pharynx length was measured using the expression area of a myo-2prom::mCherry reporter. Number of intestinal cells was scored by expression of a nuclear *elt-2prom::dsRed* reporter. Mean and range are plotted for each genotype; statistical significance was determined by an unpaired t-test, significance levels are: P>0.05 = n.s.; P<0.05 = *; P<0.01 = **; P<0.001 = ***. **H**) *pash-1(ts)* backshift experiment: miRNA-depleted embryos that developed at the restrictive temperature of 25°C and expressed mirt-35/51 individually or in combination, were transferred to the permissive temperature 16°C directly after hatching. Survival to adulthood was scored within the next 7 days.

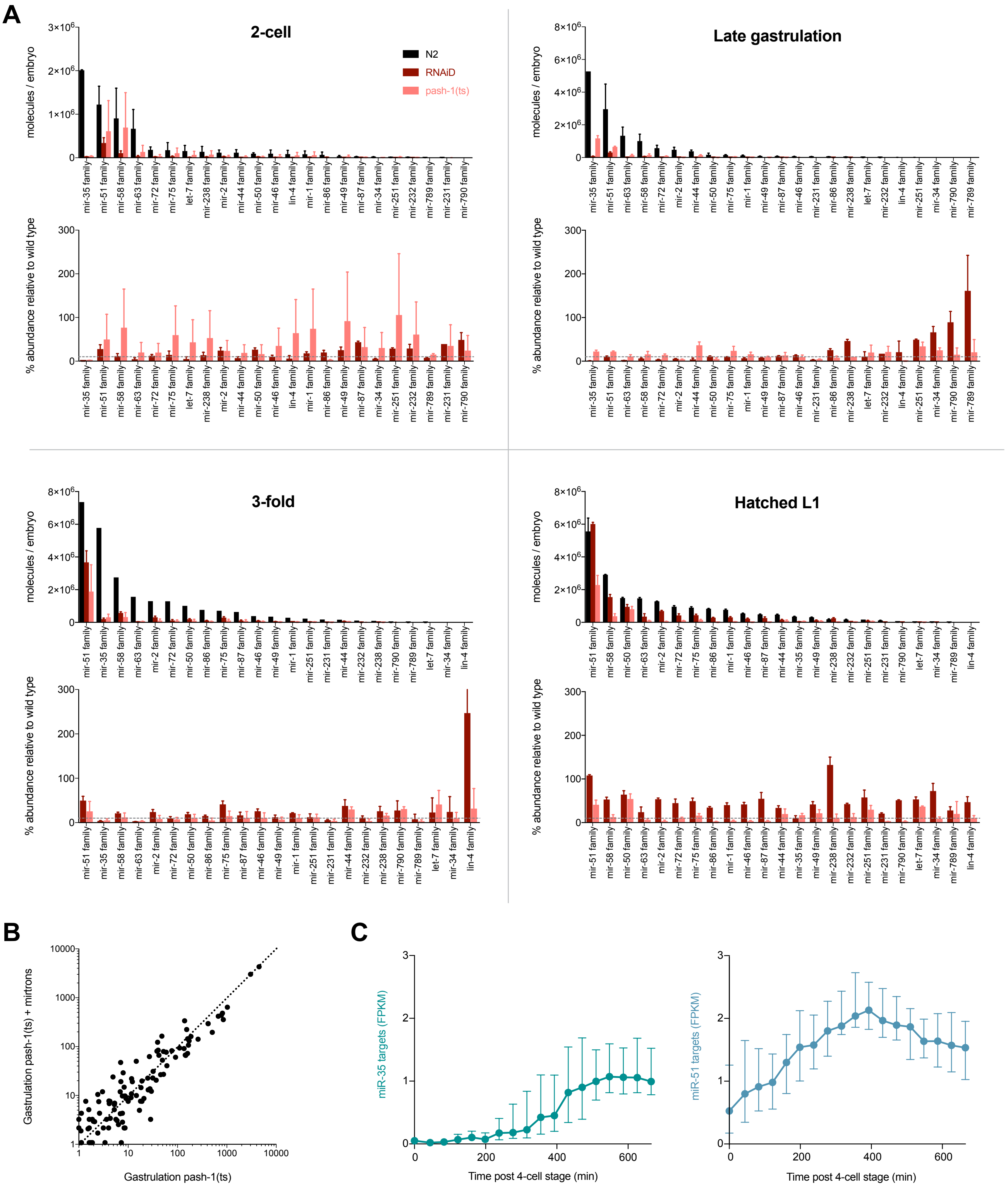

Figure S4. miRNA levels in microprocessor-depleted embryos. Related to Figure 4 and Table S2. A) For each timepoint profiled, shown are (top) the sum of the absolute abundances of canonical miRNAs grouped by families as measured by quantitative sRNA-seq (related to Figure 4A), and (bottom) the relative abundance of each miRNA family under RNAiD or pash-1(ts)-mediated depletion, in comparison to wild type. The horizontal dotted line indicates 10% of wild type. Depletion at the level of families is very strong except as noted also for individual miRNAs at the 2-cell stage in *pash-1(ts)* and at the L1 stage in RNAiD. There is no consistent leftover of other families over both depletion conditions that suggests that other miRNAs could contribute to early development. Note that the outliers in relative abundance at gastrulation and 3-fold stages correspond to families with very low absolute abundance. **B**) miRNA abundance at gastrulation in *pash-1(ts)* embryos with or without mirtrons. Mirtron-expression has no systematic effect on levels of left-over miRNAs. **C**) Embryonic expression time course of transcripts bearing conserved miR-35/51 family miRNA binding sites as predicted by TargetScan Worm 6.2 (S5, S6). Shown are median expression and 95% confidence intervals. For miR-35 family all 89 predicted targets are shown. For miR-51 family targets the top 71 expressing transcripts (out of 264 called targets in the dataset) are shown, selected based on an average FPKM value > 1 across all timepoints. The most abundant predicted miR-51^{fam} target *rps-20* (ribosomal protein, small subunit) has been removed as an outlier due to extreme abundance. Data derived from (S7).

Strain	Genotype	Comments	Figure
N2	-	wild type control strain (Brenner, 1974)	1B-D, S1B, S1F, 2B, S2E, S2G, 4A, S4A
**miR-35 family related**			
MT14533	nDf49, nDf50/mIn1 [mIs14 dpy-10(e128)] II	balanced mir-35 family deletion<br>(Alvarez-Saavedra and Horvitz, 2010)	1C, S1D, S1E, 2B, S2E
MLC812	nDf49, nDf50, II;<br>lucEx521 (mir35p::GFP_mirtron35::tbb2, myo2::mCherry)	mir-35 family deletion,<br>rescued by mirtron-35, line 1<br>generated by microinjection into MT14533	S2E
MLC825	nDf49, nDf50, II;<br>lucEx522 (mir35p::GFP_mirtron35::tbb2, myo2::mCherry)	mir-35 family deletion,<br>rescued by mirtron-35, line 2<br>generated by microinjection into MT14533	S2E
MLC826	nDf49, nDf50, II;<br>lucEx523 (mir35p::GFP_mirtron35::tbb2, myo2::mCherry)	mir-35 family deletion,<br>rescued by mirtron-35, line 3<br>generated by microinjection into MT14533	S2E
MLC1016	nDf49, nDf50, II;<br>lucIs20 (mir35p::GFP_mirtron35::tbb2, myo2::mCherry)	mir-35 family deletion,<br>rescued by mirtron-35, line 1<br>integrated via irradiation of MLC826	2B, S2E
MLC1017	nDf49, nDf50, II;<br>lucIs21 (mir35p::GFP_mirtron35::tbb2, myo2::mCherry)	mir-35 family deletion,<br>rescued by mirtron-35, line 2<br>integrated via irradiation of MLC826	S2E
MLC2353	nDf49, nDf50 / mIn1, II<br>lucEx1240 (mir35p::GFP_mirtron35(seed-mutant)::tbb2, myo2::mCherry)	balanced mir-35 family deletion,<br>expressing a seed-mutant version of mirt-35 that does not rescue<br>generated by microinjection into MT14533	S2E
MLC2354	nDf49, nDf50 / mIn1, II<br>lucEx1241 (mir35p::GFP_mirtron35(seed-mutant)::tbb2, myo2::mCherry)	balanced mir-35 family deletion,<br>expressing a seed-mutant version of mirt-35 that does not rescue<br>generated by microinjection into MT14533	S2E
MLC2355	nDf49, nDf50 / mIn1, II<br>lucEx1242 (mir35p::GFP_mirtron35(seed-mutant)::tbb2, myo2::mCherry)	balanced mir-35 family deletion,<br>expressing a seed-mutant version of mirt-35 that does not rescue<br>generated by microinjection into MT14533	S2E
**miR-51 family related**			
MLC1800	n4114, nDf67, IV; nDf58, X;<br>lucEx1057	mir-51 family deletion<br>rescued by a partially penetrant extrachromosomal array<br>expressing mir-54-56<br>generated by microinjection into MT17143	1C, S1D, S1E, 2B, S2E
MLC836	n4114, nDf67, IV/nT1 [qIS51] (IV,V); nDf58, X;<br>lucEx525 (mir52p::GFP_mirtron54.1::tbb2, myo2::mCherry)	balanced mir-51 family deletion,<br>expressing mirtron-51, line 1,<br>homozygous mutant line can be isolated,<br>generated by microinjection into MT17143	S2E
MLC839	n4114, nDf67, IV/nT1 [qIS51] (IV,V); nDf58, X;<br>lucEx528 (mir52p::GFP_mirtron54.1::tbb2, myo2::mCherry)	balanced mir-51 family deletion,<br>expressing mirtron-51, line 2,<br>homozygous mutant line can be isolated,<br>generated by microinjection into MT17143	S2E
MLC903	n4114, nDf67, IV/nT1 [qIS51] (IV,V); nDf58, X;<br>lucIs24 (mir52p::GFPstop_mirtron54.1::tbb2, myo2::mCherry)	balanced mir-51 family deletion,<br>expressing mirtron-51, line 3 (stop codon in GFP exon 1)<br>homozygous mutant line can be isolated,<br>generated by microinjection into MT17143, spontaneous integrant	2B, S2E
pash-1(ts)			
MLC860	mj100, I	pash-1(ts), strain SX1359 (Lehrbach et al., 2012),<br>outcrossed four times, lacking mjEx331	S1D-G, S2G, 3A, S3B, 4A, 4B, S4A, S4B
MLC881	mj100, I;<br>mjEx331 (eft-3p::pash-1::GFP::unc-54, myo-2p::mCherry::unc-54 3'UTR)	pash-1(ts), strains SX1359 (Lehrbach et al., 2012),<br>outcrossed four times	S1F
MLC1104	mj100, I;<br>lucIs20 (m35p::GFP_mirtron-35::tbb2, myo2::mCherry)	pash-1(ts)<br>expressing mirtron-35,	S3B, S3H
MLC1105	mj100, I;<br>lucIs24 (mir52p::GFPstop_mirtron54.1::tbb-2, elt2::dsRed, myo2::mCherry)	pash-1(ts),<br>expressing mirtron-51	S3B, S3H
MLC1795	mj100, I;<br>lucIs20 (m35p::GFP_mirtron-35::tbb2, myo2::mCherry),<br>lucIs24 (mir52p::GFPstop_mirtron54.1::tbb-2, elt2::dsRed, myo2::mCherry)	pash-1(ts)<br>expressing mirtron-35 and mirtron-51	S1G, S2G, 3A, 3B, S3E-H, 4A, S4A, S4B
**Auxin-inducible-degron**			
MLC1040	ieSi57 [eft-3p::TIR1::mRuby::unc-54 3'UTR + Cbr-unc-119(+)], II;<br>ieSi38 [sun-1p::TIR1::mRuby::sun-1 3'UTR + Cbr-unc-119(+)], IV	control strain expressing TIR-1 in soma & germline	1A, S1C, 3A
MLC1077	ieSi57 [eft-3p::TIR1::mRuby::unc-54 3'UTR + Cbr-unc-119(+)], II;<br>ieSi38 [sun-1p::TIR1::mRuby::sun-1 3'UTR + Cbr-unc-119(+)], IV;<br>lucIs20 (m35p::GFP_mirtron-35::tbb2, myo2::mCherry);<br>lucIs24 (mir52p::GFPstop_mirtron54.1::tbb-2, elt2::dsRed, myo2::mCherry)	control strain expressing TIR-1 in soma & germline<br>expressing mirtron-35 + mirtron-51	3B, S3E-G
MLC1065	luc71 (pash1::PASH-1::2xGGSG::3xFLAG::AID::myc), I;<br>ieSi57 [eft-3p::TIR1::mRuby::unc-54 3'UTR + Cbr-unc-119(+)], II;<br>ieSi38 [sun-1p::TIR1::mRuby::sun-1 3'UTR + Cbr-unc-119(+)], IV	endogenous pash-1 bearing C-terminal AID-tag = pash-1:AID<br>expressing TIR-1 in soma & germline	S1A, S1C
MLC1245	luc82 (myc::AID::3XFLAG::4xGGSG::drsh-1::4xGGSG::3xFLAG::AID::myc), I;<br>ieSi57 [eft-3p::TIR1::mRuby::unc-54 3'UTR + Cbr-unc-119(+)], II;<br>ieSi38 [sun-1p::TIR1::mRuby::sun-1 3'UTR + Cbr-unc-119(+)], IV	endogenous drsh-1 bearing N- and C-terminal AID-tag = drsh-1:AID<br>Expressing TIR-1 in soma & germline	S1A, S1C
MLC1726	luc71 (pash1::PASH-1::2xGGSG::3xFLAG::AID::myc),<br>luc82 (myc::AID::3XFLAG::4xGGSG::drsh-1::4xGGSG::3xFLAG::AID::myc), I;<br>ieSi57 [eft-3p::TIR1::mRuby::unc-54 3'UTR + Cbr-unc-119(+)], II;<br>ieSi38 [sun-1p::TIR1::mRuby::sun-1 3'UTR + Cbr-unc-119(+)], IV;	Endogenous drsh-1 and pash-1 bearing AID-tag<br>expressing TIR-1 in soma & germline	1A, S1C-E, 3A, S3A, S3B
MLC1727	luc71 (pash1::PASH-1::2xGGSG::3xFLAG::AID::myc),<br>luc82 (myc::AID::3XFLAG::4xGGSG::drsh-1::4xGGSG::3xFLAG::AID::myc), I;<br>ieSi57 [eft-3p::TIR1::mRuby::unc-54 3'UTR + Cbr-unc-119(+)], II;<br>ieSi38 [sun-1p::TIR1::mRuby::sun-1 3'UTR + Cbr-unc-119(+)], IV;<br>lucIs20 (m35p::GFP_mirtron-35::tbb2, myo2::mCherry);	endogenous drsh-1 and pash-1 bearing AID-tag<br>expressing TIR-1 in soma & germline<br>expressing mirtron-35	S3B-D
MLC1728	luc71 (pash1::PASH-1::2xGGSG::3xFLAG::AID::myc),<br>luc82 (myc::AID::3XFLAG::4xGGSG::drsh-1::4xGGSG::3xFLAG::AID::myc), I;<br>ieSi57 [eft-3p::TIR1::mRuby::unc-54 3'UTR + Cbr-unc-119(+)], II;<br>ieSi38 [sun-1p::TIR1::mRuby::sun-1 3'UTR + Cbr-unc-119(+)], IV;<br>lucIs24 (mir52p::GFPstop_mirtron54.1::tbb-2, elt2::dsRed, myo2::mCherry)	endogenous drsh-1 and pash-1 bearing AID-tag<br>expressing TIR-1 in soma & germline<br>expressing mirtron-51	S3B-D
MLC1729	luc71 (pash1::PASH-1::2xGGSG::3xFLAG::AID::myc),<br>luc82 (myc::AID::3XFLAG::4xGGSG::drsh-1::4xGGSG::3xFLAG::AID::myc), I;<br>ieSi57 [eft-3p::TIR1::mRuby::unc-54 3'UTR + Cbr-unc-119(+)], II;<br>ieSi38 [sun-1p::TIR1::mRuby::sun-1 3'UTR + Cbr-unc-119(+)], IV;<br>lucIs20 (m35p::GFP_mirtron-35::tbb2, myo2::mCherry);<br>lucIs24 (mir52p::GFPstop_mirtron54.1::tbb-2, elt2::dsRed, myo2::mCherry)	endogenous drsh-1 and pash-1 bearing AID-tag<br>expressing TIR-1 in soma & germline<br>expressing mirtron-35 + mirtron-51	1B, S2F, S2G, 3A, 3B, S3A, S3E-G, 4A, S4A
MLC2345	luc71 (pash1::PASH-1::2xGGSG::3xFLAG::AID::myc),<br>luc82 (myc::AID::3XFLAG::4xGGSG::drsh-1::4xGGSG::3xFLAG::AID::myc), I;<br>ieSi57 [eft-3p::TIR1::mRuby::unc-54 3'UTR + Cbr-unc-119(+)], II;<br>ieSi38 [sun-1p::TIR1::mRuby::sun-1 3'UTR + Cbr-unc-119(+)], IV;<br>lucIs24 (mir52p::GFPstop_mirtron54.1::tbb-2, elt2::dsRed, myo2::mCherry)<br>lucEx1235 (mir35p::GFP_mirtron35(seed-mutant)::tbb2, myo2::mCherry)	endogenous drsh-1 and pash-1 bearing AID-tag<br>expressing TIR-1 in soma & germline<br>expressing mirtron-51<br>expressing seed-mutant version of mirtron-35	S3C, S3D
**Other**			
MLC1867	nDf49, nDf50, II<br>lucEx1078 (mir35p::GFPstop_mirtron35::tbb2, myo3::mCherry, rgef1::BFP, gcy5::GFP)	control strain expressing gcy-5::GFP reporter<br>mir-35 family deletion is rescued by mirtron from ex-array	4B
MLC1907	pash-1(ts) (mj100), I<br>lucEx1078 (mir35p::GFPstop_mirtron35::tbb2, myo3::mCherry, rgef1::BFP, gcy5::GFP)<br>lucIs24 (mir52p::GFPstop_mirtron54.1::tbb-2, elt2::dsRed, myo2::mCherry)	pash-1(ts)<br>expressing mirtron-35 and mirtron-51<br>expressing gcy-5::GFP reporter to assess lsy-6 phenotype	4B
MLC1926	lsy-6 (ot71), V<br>lucEx1078 (mir35p::GFPstop_mirtron35::tbb2, myo3::mCherry, rgef1::BFP, gcy5::GFP)	lsy-6(0)<br>expressing gcy-5::GFP reporter to assess lsy-6 phenotype	4B

Table S1. Strains used and generated in this study. Related to STAR Methods. Name, genotype, and origin of strains as well as corresponding figures are denoted in the respective columns. Key strains are deposited with the CGC, other strains are available upon request.

Supplemental References

S1. W. J. Chung et al. Computational and experimental identification of mirtrons in Drosophila melanogaster and Caenorhabditis elegans. Genome Research. 21, 286–300 (2011).

S2. N. R. Markham, M. Zuker. DINAMelt web server for nucleic acid melting prediction. Nucleic Acids Research. 33, W577–81 (2005).

S3. M. M. Reimão-Pinto et al. Uridylation of RNA Hairpins by Tailor Confines the Emergence of MicroRNAs in Drosophila. Molecular Cell. 59, 203–216 (2015).

S4. D. Bortolamiol-Becet et al. Selective Suppression of the Splicing-Mediated MicroRNA Pathway by the Terminal Uridyltransferase Tailor. Molecular Cell. 59, 217–228 (2015).

S5. B. P. Lewis, C. B. Burge, D. P. Bartel. Conserved seed pairing, often flanked by adenosines, indicates that thousands of human genes are microRNA targets. Cell. 120, 15–20 (2005).

S6. C. H. Jan, R. C. Friedman, J. G. Ruby, D. P. Bartel. Formation, regulation and evolution of Caenorhabditis elegans 3′UTRs. Nature. 469, 97–101 (2011).

S7. M. E. Boeck et al. The time-resolved transcriptome of C. elegans. Genome Research. 26, 1441–1450 (2016).
