# Supplementary material for: Two MicroRNAs Are Sufficient for Embryonic Patterning in C. elegans
Source: Curr Biol. 2020 Dec 21;30(24):5058–5065.e5. doi: 10.1016/j.cub.2020.09.066 (PMC7758728; doi:10.1016/j.cub.2020.09.066)
Supplement: Document S2. Article plus Supplemental Information [file mmc3.pdf]

Report

Current Biology

Two MicroRNAs Are Sufficient for Embryonic Patterning in *C. elegans*

Graphical Abstract

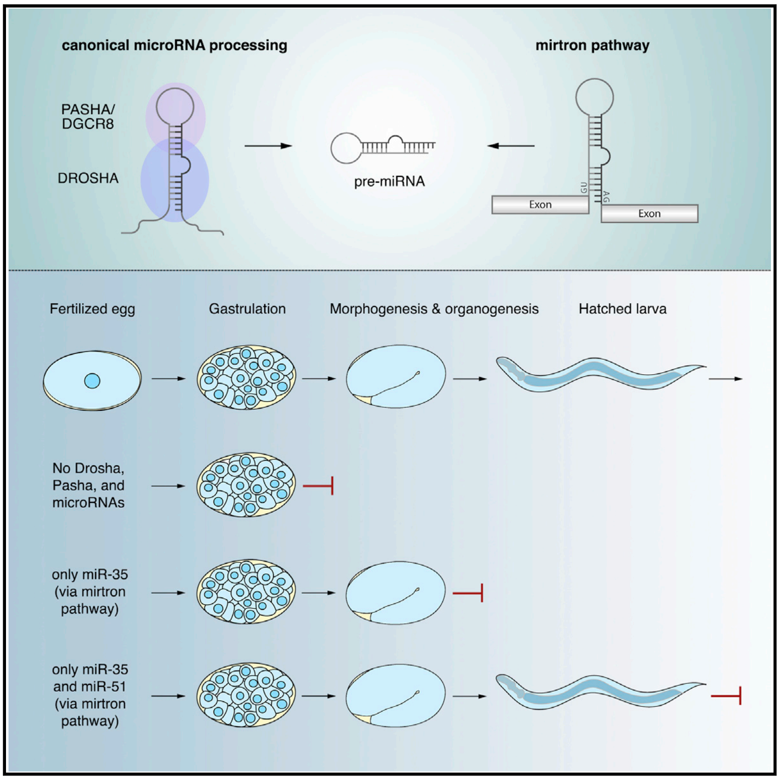

Highlights

- Depletion of Drosha and Pasha results in embryos that fail to undergo morphogenesis
- The mirtron pathway enables expression of miRNAs in the absence of Drosha and Pasha
- Two miRNAs are sufficient to rescue embryogenesis in the absence of Drosha and Pasha
- miR-35 and miR-51 play an unexplored, likely conserved role in animal development

Authors

Philipp J. Dexheimer, Jingkui Wang, Luisa Cochella

Correspondence

cochella@imp.ac.at

In Brief

Dexheimer et al. show that depletion of maternal and zygotic Drosha and Pasha in *C. elegans* embryos causes failure to undergo morphogenesis. This can be overcome by re-introducing two miRNAs, miR-35 and miR-51, resulting in morphologically normal larvae. These conserved miRNAs account for the essential embryonic function of Drosha and Pasha.

Dexheimer et al., 2020, Current Biology *30*, 5058–5065
December 21, 2020 © 2020 The Author(s). Published by Elsevier Inc.
https://doi.org/10.1016/j.cub.2020.09.066

CellPress
OPEN ACCESS

Current Biology

Report

Two MicroRNAs Are Sufficient for Embryonic Patterning in *C. elegans*

Philipp J. Dexheimer,[1] Jingkui Wang,[1] and Luisa Cochella[1,2,*]
[1]Research Institute of Molecular Pathology (IMP), Vienna BioCenter (VBC), Campus-Vienna-Biocenter 1, 1030 Vienna, Austria
[2]Lead Contact
*Correspondence: cochella@imp.ac.at
https://doi.org/10.1016/j.cub.2020.09.066

SUMMARY

MicroRNAs (miRNAs) are a class of post-transcriptional repressors with diverse roles in animal development and physiology [1]. The Microprocessor complex, composed of Drosha and Pasha/DGCR8, is necessary for the biogenesis of all canonical miRNAs and essential for the early stages of animal embryogenesis [2–8]. However, the cause for this requirement is largely unknown. Animals often express hundreds of miRNAs, and it remains unclear whether the Microprocessor is required to produce one or few essential miRNAs or many individually non-essential miRNAs. Additionally, both Drosha and Pasha/DGCR8 bind and cleave a variety of non-miRNA substrates [9–15], and it is unknown whether these activities account for the Microprocessor's essential requirement. To distinguish between these possibilities, we developed a system in *C. elegans* to stringently deplete embryos of Microprocessor activity. Using a combination of auxin-inducible degradation (AID) and RNA interference (RNAi), we achieved Drosha and Pasha/DGCR8 depletion starting in the maternal germline, resulting in Microprocessor and miRNA-depleted embryos, which fail to undergo morphogenesis or form organs. Using a Microprocessor-bypass strategy, we show that this early embryonic arrest is rescued by the addition of just two miRNAs, one miR-35 and one miR-51 family member, resulting in morphologically normal larvae. Thus, just two out of ~150 canonical miRNAs are sufficient for morphogenesis and organogenesis, and the processing of these miRNAs accounts for the essential requirement for Drosha and Pasha/DGCR8 during the early stages of *C. elegans* embryonic development.

RESULTS

The Microprocessor Is Necessary for Morphogenesis and Organogenesis in *C. elegans*

MicroRNAs (miRNAs) are short non-coding RNAs that act with Argonaute proteins to elicit translational repression and decay of specific target mRNAs [1]. miRNAs are excised from longer genome-encoded transcripts by the Microprocessor, a complex of the endonuclease Drosha and its partner Pasha/DGCR8, and further processed by another endonuclease, Dicer [1]. Loss of the miRNA processing machinery or the Argonaute effector proteins results in early developmental lethality in every animal species studied [16], but the cause for this strict requirement remains unclear. In zebrafish, the abundant miR-430 accounts for a large fraction of the embryonic defects observed upon loss of Dicer [17], acting at least in part by clearing maternal mRNAs [18]. However, miR-430 is only present in vertebrates, and in zebrafish in particular, it forms an unusual cluster with >50 genomic copies [19]. In other animals, the reasons for the essentiality of the miRNA pathway during early embryonic development remain unknown.

To investigate the role of the Microprocessor during embryogenesis of the nematode *C. elegans*, we developed a conditional approach for stringent removal of Drosha and Pasha/DGCR8 and thus miRNAs. Microprocessor depletion in embryos is generally challenging, given the maternal contribution of mRNA and protein. In *C. elegans* in particular, the function of the Microprocessor has not been studied in embryos because homozygous Drosha (*drsh-1*) or Pasha/DGCR8 (*pash-1*) mutants derived from heterozygous mothers carry enough maternal RNA and/or protein to develop into adults, albeit sterile [2, 3]. To overcome this, we took advantage of the *Arabidopsis* auxin inducible degradation (AID) system [20]: we tagged endogenous Drosha (both at the N and C termini) and Pasha/DGCR8 (at the C terminus) with the TIR1 ubiquitin ligase-recognition peptide and expressed TIR1 in both germline and soma to clear maternal and zygotic tagged proteins (Figures 1A and S1A). To further reduce Microprocessor levels, we simultaneously triggered systemic RNAi against *pash-1* by feeding (Figure S1B). To ensure elimination of maternal Microprocessor and its products, mothers were grown on auxin and RNAi-eliciting bacteria for 20 h before harvesting just-fertilized embryos (2-cell stage). This treatment caused an increase in unfertilized oocytes and a concomitant reduction in the number of embryos produced, as expected based on the described roles for miRNAs in germline function [2, 3, 21–23]. However, we could still harvest enough 2-cell embryos and follow their development. Whereas depletion of either PASH-1 or DRSH-1 alone was sufficient to induce penetrant embryonic lethality (Figure S1C), simultaneous removal of both proteins and *pash-1* mRNA resulted in the earliest, most homogeneous arrest phenotype (Figures S1C–S1E). We refer to this combined AID and RNAi manipulation as RNAiD. This system

 © 2020 The Author(s). Published by Elsevier Inc.
This is an open access article under the CC BY license (http://creativecommons.org/licenses/by/4.0/).

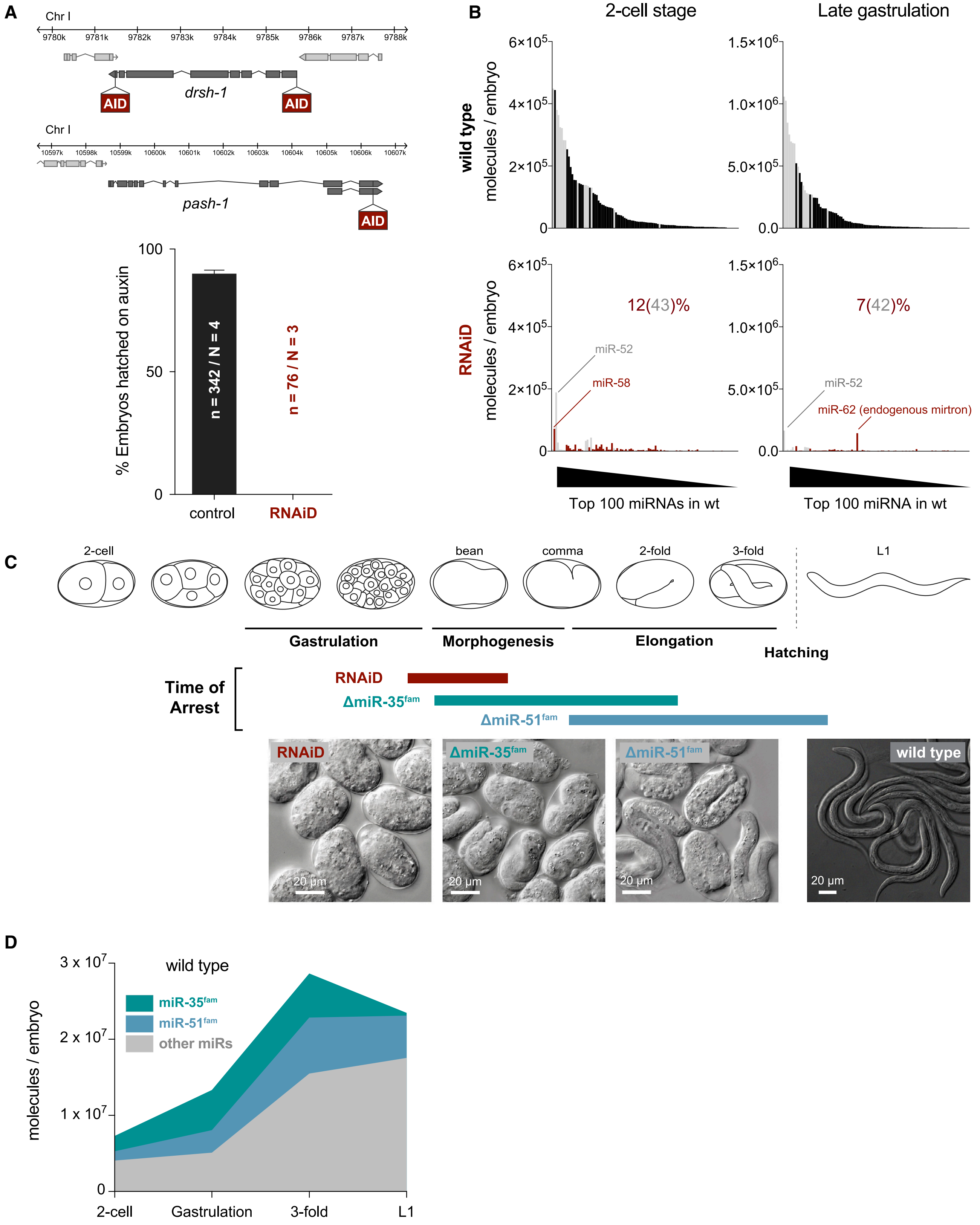

(legend on next page)

enabled examination of *C. elegans* embryos that were largely depleted of maternal and zygotic miRNAs (Figure 1B). 100% of these embryos arrest uniformly at the end of gastrulation/onset of morphogenesis, lacking distinguishable internal structure other than the intestine primordium, and eventually die (Figures 1A, 1C, S1D, and S1E).

To deplete the Microprocessor by independent means, we used *pash-1(mj100)*, a temperature-sensitive allele of Pasha/DGCR8 [24]. Shifting mothers to the restrictive temperature 7 h before harvesting 2-cell embryos (the maximum possible before the mothers visibly deteriorate) also resulted in fully penetrant embryonic lethality (Figure S1F). However, the timing of arrest was variable (Figures S1D and S1E), consistent with the observation that an average of 35% of maternal miRNA content remained at the 2-cell stage (Figure S1G). Thus, although the *pash-1(ts)* allele is efficient for removing miRNAs from gastrulation onward, RNAiD results in stringent miRNA depletion from oogenesis up to the time of embryonic arrest.

In *C. elegans,* no individual miRNA is known to be essential for embryogenesis, but two families with multiple, redundant members each, miR-35^{fam} and miR-51^{fam}, are necessary at two distinct time points [25–27]. Deletion of all eight members of the miR-35^{fam} (miR-35-42) causes arrest after completing gastrulation during early morphogenesis, -whereas animals lacking the six miR-51^{fam} members (miR-51-56) have later elongation and organogenesis defects and arrest as late embryos or malformed larvae (Figures 1C, S1D, and S1E). These two families constitute ~50% of the early embryo miRNA content (Figure 1D), and they are broadly if not ubiquitously expressed during embryogenesis [26, 27]. The remaining >130 miRNAs are individually much less abundant and tend to be expressed in cell-type-specific manner [16, 28, 29].

The effect of RNAiD was more severe than the arrest phenotype of animals lacking the early-acting miR-35^{fam} (Figures 1C, S1D, and S1E), raising the possibility that other miRNAs could be necessary for earlier stages of embryogenesis. Alternatively, non-miRNA-related activities of Drosha and Pasha/DGCR8 could be responsible for the more severe phenotype upon RNAiD. Moreover, it is possible that the combined loss of miR-35^{fam} and miR-51^{fam} accounts for the observed defects in embryogenesis in the absence of the Microprocessor.

The Mirtron Pathway Can Be Used to Produce Microprocessor-Independent miRNAs *In Vivo*

To distinguish between the possibilities raised above, we asked whether miR-35^{fam} and miR-51^{fam} are sufficient for embryogenesis in the absence of the Microprocessor and all other miRNAs. As every individual member of the miR-35 or miR-51 family can rescue the complete deletion of the respective family [26], we reasoned that we could re-introduce one member of each family into Microprocessor-depleted animals to test their sufficiency. Previous approaches to introduce miRNAs in a Microprocessor-independent manner relied on injection of processed RNA duplexes [17]. Instead, we developed a transgenic strategy for Microprocessor-independent miRNA delivery, which recapitulates the spatiotemporal specificity of expression of re-introduced miRNAs. Specifically, we designed miR-35^{fam} and miR-51^{fam} mirtrons, which are processed by the spliceosome and are subsequently cleaved by Dicer to produce mature miRNAs (Figures 2A and S2) [30, 31].

Mirtron-35 and mirtron-51 (mirt-35/51) were expressed under their respective endogenous promoters. Each mirtron was able to largely rescue embryonic lethality of the corresponding miRNA family deletion (to 85% and 75%, respectively; Figures 2B and S2E). In contrast, a mirt-35 variant with a mutated seed sequence failed to overcome lethality caused by deletion of miR-35^{fam} (Figure S2E), ruling out a non-specific rescue by mirt-35 due to its sheer abundance and reported interaction of miR-35^{fam} with other small RNA pathways [32].

Small RNA sequencing from animals expressing the mirtrons in Microprocessor-depleted backgrounds revealed that each mirtron was processed accurately into a mature miRNA with the expected 5′ end, preserving the family seed sequence and thus target specificity (Figure S2F) [33]. Both mirtrons displayed heterogeneity at the 3′ end: 25%–45% of mirt-35 had non-templated addition of a single nucleotide, uridine in >90% of cases, consistent with the described tailing of mirtrons in *Drosophila* [34, 35], and 90%–95% of mirt-51 was trimmed by 1 nt, reducing its length from 24 to 23 nt. The mirtrons were practically undetectable in 2-cell embryos, consistent with commonly observed transgene silencing in the *C. elegans* germline. However, correctly processed mirtrons reached levels comparable to those of the respective families at gastrulation and later time points (Figure S2G). This establishes the mirtron pathway as an efficient tool to express Microprocessor-independent miRNAs that substitute for abundant, endogenous miRNAs.

Two miRNAs Rescue Morphogenesis and Organogenesis in Microprocessor-Depleted Embryos

Simultaneous expression of mirt-35 and mirt-51 restored the ability of the RNAiD and *pash-1(ts)* Microprocessor-deficient embryos to produce morphologically normal larvae in ~50% of cases (Figure 3), out of a theoretical maximum of ~60%, given the imperfect rescue of each of the mirtron transgenes (Figure 2B). The remaining ~50% of embryos showed variable levels of rescue, with ~70% reaching later stages of development, albeit with morphogenesis defects (Figure S3A). Both mirtrons were necessary to rescue embryogenesis up to successful

Figure 1. miRNAs Are Essential for Morphogenesis and Organogenesis

(A) Schematic of the genetic setup to deplete the Microprocessor via the AID system and hatching rate of control (expressing only TIR1) or Microprocessor-depleted embryos by RNAiD. n, number of embryos scored; N, number of independent experiments. Error bars represent the standard error of the proportion.

(B) Absolute miRNA abundance in WT and RNAiD embryos at the 2-cell stage and at the end of gastrulation as determined by quantitative small RNA sequencing. The 100 most abundant miRNAs in WT embryos at each stage are shown. Remaining miRNAs of the miR-35 and miR-51 families are depicted in gray. The percentage of miRNA molecules remaining relative to WT is indicated, with relative contributions of miR-35 and miR-51 families combined noted in brackets. Note that the samples analyzed here are the same as in Figures S2F, S2G, 4A, and S4A.

(C) Schematic of the predominant time of arrest and representative images of embryos of the indicated genotypes.

(D) Absolute miRNA abundance in developing WT embryos as measured by small RNA-seq, highlighting the proportion of miR-35 and miR-51 families.

See also Figure S1.

CellPress
OPEN ACCESS

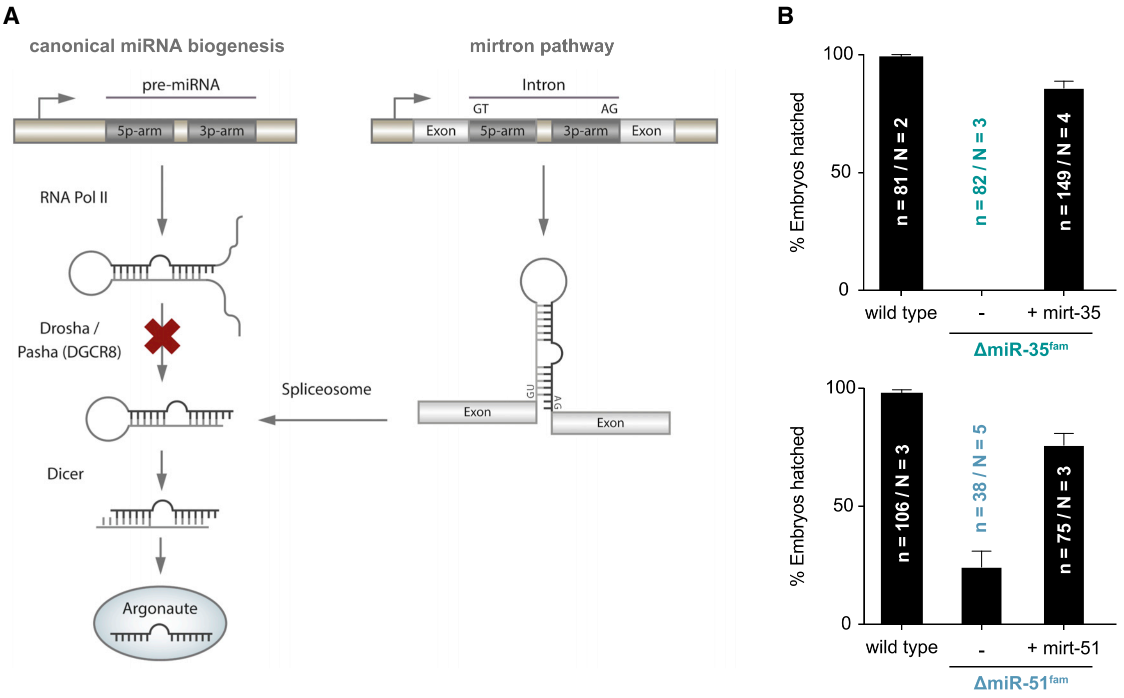

Figure 2. The Mirtron Pathway Can Substitute for Abundant Endogenous miRNAs

(A) Schematic of the experimental strategy to test for miRNA sufficiency: a block in miRNA biogenesis at the Microprocessor level is bypassed by splicing during the process of mirtron biogenesis.

(B) Hatching rates of miR-35^{fam} and miR-51^{fam} mutant embryos with or without mirt-35 or mirt-51, respectively. Error bars represent the standard error of the proportion.

See also Figure S2.

hatching in RNAiD-treated animals. However, in the *pash-1(ts)* background, in which a higher fraction of maternal miR-35^{fam} and miR-51^{fam} members persists in early embryos (Figure S1G), each mirtron provided partial rescue on its own (Figure S3B). Consistent with the earlier role of miR-35^{fam}, RNAiD embryos expressing just mirt-35 overcame the early arrest phenotype but failed later during elongation predominantly as malformed 2- to 3-fold embryos (Figures S3C and S3D). Notably, embryos expressing only mirt-51 developed a little further than RNAiD embryos without mirtrons. This suggests that, in addition to a role at later stages of embryogenesis, miR-51^{fam} has a contribution early on, which can be observed in the absence of other miRNAs. We thus conclude that the more severe phenotype of RNAiD relative to miR-35^{fam} mutants is due to the action of other miRNAs, primarily of miR-51^{fam}. In addition, RNAiD embryos expressing mirt-51 together with the seed mutant version of mirt-35 looked indistinguishable from embryos expressing mirt-51 alone (Figures S3C and S3D). These results indicate that embryogenesis requires the activity of a miR-35 and a miR-51 family member and not any abundant miRNA that might either balance other small RNA pathways, prevent non-specific loading of Argonaute proteins, or act through distinct indirect mechanisms [32].

Mirtron-rescued larvae formed all major organs and appeared morphologically normal, although they were slightly shorter and wider (Figures 3B and S3E–S3G), likely due to the lack of miR-58^{fam} [26]. They also crawled and fed, indicating that the production of these two miRNAs accounts for the majority of the embryogenesis defects observed in Microprocessor-deficient *C. elegans*. However, rescued larvae rarely molted and all died. To test whether these larvae could resume development upon regaining the ability to produce miRNAs, we shifted mirtron-rescued *pash-1(ts)* L1 larvae back to the permissive temperature. This enabled a quarter of the larvae to become fertile adults, suggesting that L1 larvae rescued by mirt-35 and mirt-51 during embryogenesis are competent to complete development (Figure S3H) and that additional miRNAs are required for larval progression (in addition to *lin-4* and *let-7*, whose loss causes specific defects distinct from those observed here) [36, 37].

The ability to produce rescued larvae enabled us to assess the level of miRNA depletion beyond the gastrulation time point. We performed quantitative small RNA sequencing in 3-fold embryos (8 h post-2-cell) and in hatched L1 larvae, in addition to the 2-cell and late gastrulation time points (Figures 4A and S4A). Note that the depletion data plotted in Figure 1B are part of this full time course obtained from mirtron-expressing embryos, as we determined that mirtron expression did not affect the levels of other miRNAs under Microprocessor depletion conditions (Figure S4B). Mirtron-rescued 3-fold embryos and L1 larvae of the *pash-1(ts)* strain were strongly depleted of miRNAs (14% and 20% of miRNA molecules remained compared to wild type [WT], of which 54% and 51% were miR-35/51^{fam} members, respectively). Notably, two miRNAs persisted, the miR-51^{fam} member miR-52, which is partially resistant to Microprocessor

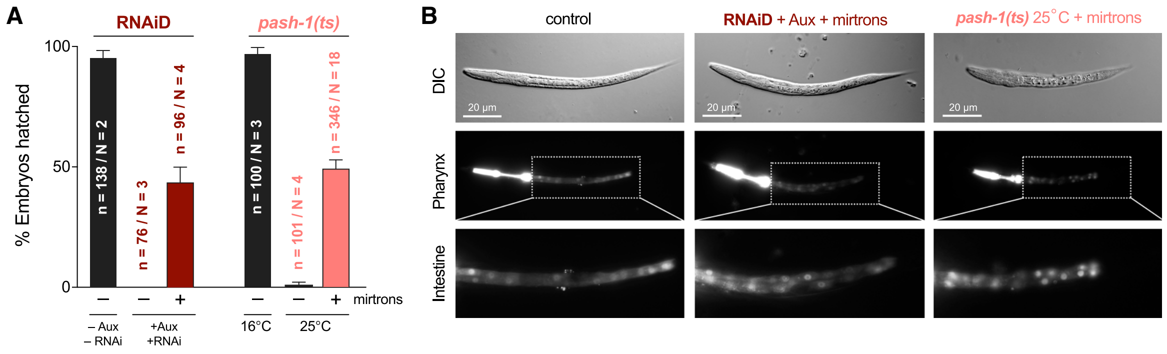

Figure 3. Two miRNAs Are Sufficient for Embryonic Patterning in the Absence of Drosha and Pasha

(A) Hatching rates of RNAiD or *pash-1(ts)* Microprocessor-depleted animals, with or without mirt-35 and mirt-51. Error bars represent the standard error of the proportion.

(B) Representative images of mirtron-rescued L1 larvae. Shown are differential interference contrast (DIC)/Nomarski images, fluorescently labeled pharynx (*myo-2^{prom}::mCherry*), and intestine (*elt-2^{prom}::NLS-dsRed*). For comparison to arrested embryos without mirtrons, see Figures 1A and S1D.

See also Figure S3.

ablation, and miR-62, an endogenous mirtron without obvious function [25]. RNAiD treatment, which was very efficient for depletion of maternal and zygotic miRNAs up to the end of gastrulation, still caused significant miRNA depletion in 3-fold embryos (27% remaining, of which 51% correspond to miR-35/51^{fam} members) but was less effective for miRNA depletion in L1 larvae, likely because auxin levels in late embryos become insufficient to trigger efficient degradation. From the combination of the RNAiD and the *pash-1(ts)* experiments, we conclude that miR-35^{fam} and miR-51^{fam} are sufficient for embryonic development in the absence of Drosha and Pasha/DGCR8 activities.

We also confirmed that mirtron-rescued larvae were functionally depleted of miRNAs that act later in cell-type-specific manners. For instance, *lsy-6* is a miRNA necessary to lateralize the ASE pair of neurons [38]. Mirtron-rescued *pash-1(ts)* larvae behaved identically to larvae bearing a genomic deletion of *lsy-6* in their inability to properly specify the ASE lateral asymmetry (Figure 4B). Hence, these larvae, which lack practically all miRNAs except for miR-35/51^{fam}, also provide an opportunity to explore the global contribution of such later-acting, cell-type-specific miRNAs.

DISCUSSION

We showed that Microprocessor-depleted *C. elegans* embryos arrest shortly after gastrulation, failing to undergo morphogenesis or to form organs. This arrest phenotype is earlier than in Dicer-deficient zebrafish, which manage to form most organs, albeit imperfectly [17]. The early arrest is reminiscent of Pasha/DGCR8 mutant mouse embryos, which die during gastrulation [5]. Two conserved miRNAs, one member of each miR-35^{fam} and miR-51^{fam}, are sufficient to overcome this arrest in *C. elegans*.

Both Drosha and Pasha/DGCR8 bind a variety of non-miRNA substrates [9–11] and perform regulatory functions through cleavage or binding of mRNAs [12–15]. However, the biological significance of these functions remained unclear. As embryonic lethality caused by absence of Pasha and Drosha is rescued by addition of miR-35 and miR-51, we conclude that neither protein carries out essential miRNA-independent functions during early *C. elegans* embryogenesis.

The miR-35^{fam} and miR-51^{fam} are broadly conserved: miR-35^{fam} is found across Protostomia and miR-51^{fam} is part of the miR-100^{fam}, one of the most conserved families across all animals [27, 39]. Yet the functions of these two miRNA families remain poorly understood. In *C. elegans*, miR-35^{fam} has been implicated in fecundity, sex determination, and apoptosis [23, 40–43]. However, none of these functions explain the fully penetrant embryonic lethality. The role of miR-51^{fam} remains less explored; part of the defects observed in *C. elegans* embryos lacking this family are related to the fat cadherin ortholog, CDH-3 [27]. However, deregulation of CDH-3 does not explain the embryonic lethality in the absence of miR-51^{fam}.

Zebrafish miR-430 is expressed in the zygote, where it plays a role in maternal mRNA clearance [18]. In contrast, miR-35^{fam} and miR-51^{fam} are present in the germline, and at least for miR-35^{fam}, there is evidence that it is active in the maternal germline [42, 43]. Moreover, the levels of the predicted targets of the miR-35^{fam} and miR-51^{fam} tend to stay constant during embryogenesis or even increase (Figure S4C) [44]. These observations strongly suggest that miR-35^{fam} and miR-51^{fam} do not function in maternal mRNA clearance. These two miRNA families thus play a yet unidentified, likely conserved function in animal embryogenesis.

We propose that the finding that two miRNAs are sufficient for early embryonic patterning in *C. elegans*, together with the predominance of miR-430 in zebrafish embryogenesis [17], reflects a more general principle of miRNA function in animal development, namely that only few miRNAs have acquired functions in the early stages of embryogenesis. Notably, these miRNAs with early, essential functions are present as families with multiple members that share the same seed sequence and are expressed broadly and abundantly. Consistently, in the mouse, the only miRNAs known so far to play a role in early patterning are the miR-290 and miR-302 clusters [45]; deletion of both clusters simultaneously causes arrest around E6.5, similar to the loss of DGCR8 [5].

Whereas few miRNAs act at early stages of embryogenesis, we propose that the majority of miRNAs in *C. elegans*, but also in other animals, play roles in the development and physiology

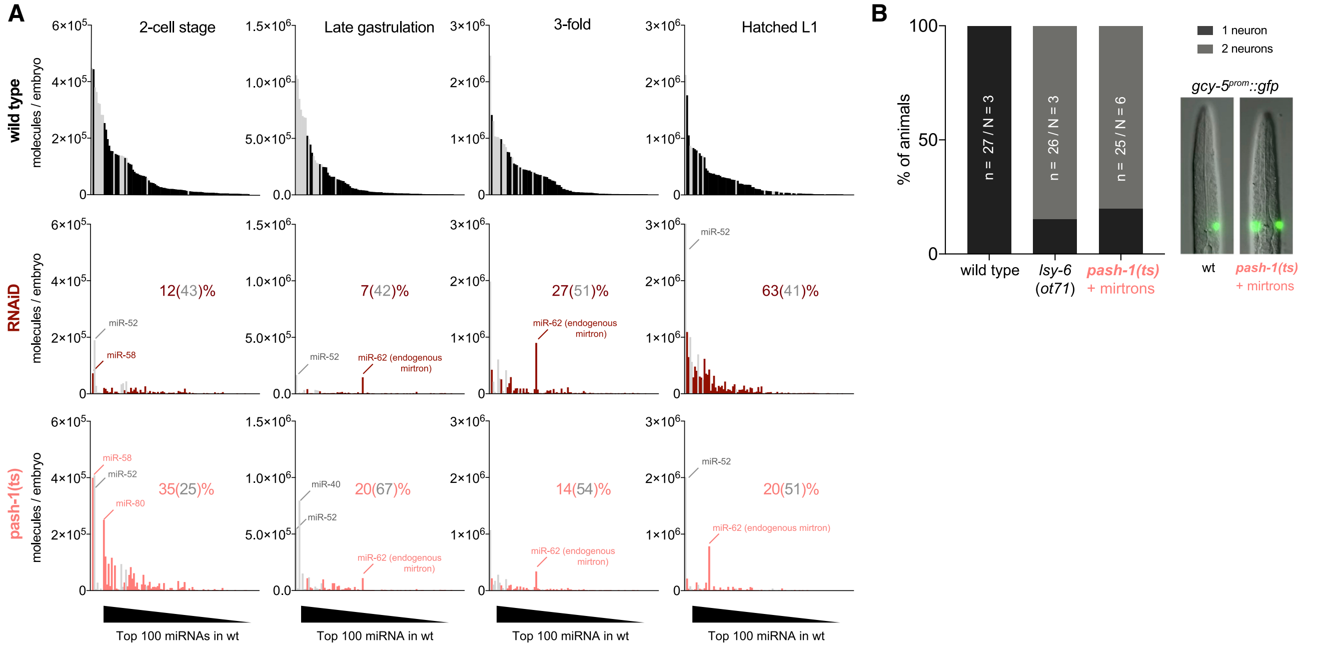

Figure 4. Mirtron-Rescued Embryos Lack Canonical miRNAs at the Molecular and Functional Level

(A) Absolute miRNA abundance in WT, RNAiD, and *pash-1(ts)* animals under restrictive conditions, at the indicated time points, measured by spike-in normalized small RNA-seq as in Figure 1B. Shown are the top 100 miRNAs, ranked by abundance in WT samples. Remaining miRNAs of the miR-35 and -51 families are in gray. For comparison, data for 2-cell and gastrulation time points are replotted from Figures 1B and S1G. The percentage of miRNA molecules remaining relative to WT is indicated, with the relative contribution of mir-35 and mir-51 families combined noted in brackets. For quantification of miRNAs grouped by families, see Figure S4A.

(B) Functional readout for the function of the late embryonic miRNA *lsy-6*. In WT animals, only the right ASE neuron expresses *gcy-5:gfp*, but absence of functional *lsy-6* results in activation of the reporter in both the left and right neurons.

of specialized cells. Most miRNAs in *C. elegans* are expressed with high cell type specificity at later stages of embryogenesis and/or in differentiated cells [16, 28, 29]. Although not strictly required for survival under laboratory conditions, such cell-specific miRNAs contribute to the acquisition of the phenotypic diversity that is critical for the animal's evolutionary success in complex natural environments (e.g., by enabling development and function of diverse sensory neurons) [38, 46]. Similarly, many miRNAs in other animals are expressed with high cell type specificity [47, 48] and, based on a comprehensive survey in the mouse [1], seem to function in cell- or tissue-specific development or physiology. Many of these mouse miRNAs are also essential for viability, although lethality in these cases occurs late in development in correctly patterned embryos that fail to generate specific cell types, e.g., miR-1-deficient mice die shortly after birth due to cardiac muscle defects [49]. In contrast in *C. elegans*, most miRNAs are not required for viability in the lab, even at later stages [25]. This distinction between worms and mice likely reflects a difference in the tolerance of each organism to defects in the production of differentiated cells rather than generally distinct roles of miRNAs (e.g., miR-1-deficient *C. elegans* also show a range of molecular and cellular defects in muscles, but the animals are still fully viable in the lab) [50–52].

Our work supports the distinction of two classes of miRNAs that contribute to the production of a multicellular organism in different ways: few miRNAs act in the early stages of development, whereas the majority of miRNAs play more specialized roles at later stages [16]. This classification may provide a more general framework for understanding the contributions of miRNAs to animal development and evolution.

STAR★METHODS

Detailed methods are provided in the online version of this paper and include the following:

- KEY RESOURCES TABLE
- RESOURCE AVAILABILITY
  - Lead Contact
  - Materials Availability
  - Data and Code Availability
- EXPERIMENTAL MODEL AND SUBJECT DETAILS
- METHOD DETAILS
  - Mirtron design and expression
  - Genome engineering
  - Pash-1(ts) experiments
  - RNAiD experiments
  - Western blotting
  - Hatching assays
  - Microscopy
  - Small RNA sequencing
- QUANTIFICATION AND STATISTICAL ANALYSIS

SUPPLEMENTAL INFORMATION

Supplemental Information can be found online at https://doi.org/10.1016/j.cub.2020.09.066.

A video abstract is available at https://doi.org/10.1016/j.cub.2020.09.066#mmc4.

ACKNOWLEDGMENTS

We thank Dr. Katherine McJunkin and Cochella lab members for feedback on the manuscript, Guy Riddihough (Life Science Editors) for editing, and Mila Asparuhova and Katharina Umundum for generation of transgenic animals. We also thank WormBase. Some strains were provided by the CGC, which is funded by NIH Infrastructure Program P40 OD010440. This work was supported by grant ERC-StG-337161 (FP7/2007-2013) from the European Research Council and grants SFB-F43-23 and W-1207-B09 from the Austrian Science Fund to L.C. Basic research at IMP is supported by Boehringer Ingelheim.

AUTHOR CONTRIBUTIONS

P.J.D. and L.C. designed the experiments, wrote the manuscript, and prepared the figures; P.J.D. performed wet-lab experiments; J.W. conducted bioinformatic analyses.

DECLARATION OF INTERESTS

The authors declare no competing interests.

Received: July 8, 2020
Revised: August 25, 2020
Accepted: September 21, 2020
Published: October 29, 2020

REFERENCES

1. Bartel, D.P. (2018). Metazoan microRNAs. Cell *173*, 20–51.
2. Denli, A.M., Tops, B.B.J., Plasterk, R.H.A., Ketting, R.F., and Hannon, G.J. (2004). Processing of primary microRNAs by the Microprocessor complex. Nature *432*, 231–235.
3. Rios, C., Warren, D., Olson, B., and Abbott, A.L. (2017). Functional analysis of microRNA pathway genes in the somatic gonad and germ cells during ovulation in C. elegans. Dev. Biol. *426*, 115–125.
4. Martin, R., Smibert, P., Yalcin, A., Tyler, D.M., Schäfer, U., Tuschl, T., and Lai, E.C. (2009). A Drosophila pasha mutant distinguishes the canonical microRNA and mirtron pathways. Mol. Cell. Biol. *29*, 861–870.
5. Wang, Y., Medvid, R., Melton, C., Jaenisch, R., and Blelloch, R. (2007). DGCR8 is essential for microRNA biogenesis and silencing of embryonic stem cell self-renewal. Nat. Genet. *39*, 380–385.
6. Gregory, R.I., Yan, K.-P., Amuthan, G., Chendrimada, T., Doratotaj, B., Cooch, N., and Shiekhattar, R. (2004). The Microprocessor complex mediates the genesis of microRNAs. Nature *432*, 235–240.
7. Han, J., Lee, Y., Yeom, K.-H., Kim, Y.-K., Jin, H., and Kim, V.N. (2004). The Drosha-DGCR8 complex in primary microRNA processing. Genes Dev. *18*, 3016–3027.
8. Song, J.L., Stoeckius, M., Maaskola, J., Friedländer, M., Stepicheva, N., Juliano, C., Lebedeva, S., Thompson, W., Rajewsky, N., and Wessel, G.M. (2012). Select microRNAs are essential for early development in the sea urchin. Dev. Biol. *362*, 104–113.
9. Macias, S., Plass, M., Stajuda, A., Michlewski, G., Eyras, E., and Cáceres, J.F. (2012). DGCR8 HITS-CLIP reveals novel functions for the Microprocessor. Nat. Struct. Mol. Biol. *19*, 760–766.
10. Kim, B., Jeong, K., and Kim, V.N. (2017). Genome-wide mapping of DROSHA cleavage sites on primary microRNAs and noncanonical substrates. Mol. Cell *66*, 258–269.e5.
11. Gromak, N., Dienstbier, M., Macias, S., Plass, M., Eyras, E., Cáceres, J.F., and Proudfoot, N.J. (2013). Drosha regulates gene expression independently of RNA cleavage function. Cell Rep. *5*, 1499–1510.
12. Han, J., Pedersen, J.S., Kwon, S.C., Belair, C.D., Kim, Y.-K., Yeom, K.-H., Yang, W.Y., Haussler, D., Blelloch, R., and Kim, V.N. (2009). Posttranscriptional crossregulation between Drosha and DGCR8. Cell *136*, 75–84.
13. Triboulet, R., Chang, H.-M., Lapierre, R.J., and Gregory, R.I. (2009). Post-transcriptional control of DGCR8 expression by the Microprocessor. RNA *15*, 1005–1011.
14. Kadener, S., Rodriguez, J., Abruzzi, K.C., Khodor, Y.L., Sugino, K., Marr, M.T., 2nd, Nelson, S., and Rosbash, M. (2009). Genome-wide identification of targets of the drosha-pasha/DGCR8 complex. RNA *15*, 537–545.
15. Knuckles, P., Vogt, M.A., Lugert, S., Milo, M., Chong, M.M.W., Hautbergue, G.M., Wilson, S.A., Littman, D.R., and Taylor, V. (2012). Drosha regulates neurogenesis by controlling neurogenin 2 expression independent of microRNAs. Nat. Neurosci. *15*, 962–969.
16. Alberti, C., and Cochella, L. (2017). A framework for understanding the roles of miRNAs in animal development. Development *144*, 2548–2559.
17. Giraldez, A.J., Cinalli, R.M., Glasner, M.E., Enright, A.J., Thomson, J.M., Baskerville, S., Hammond, S.M., Bartel, D.P., and Schier, A.F. (2005). MicroRNAs regulate brain morphogenesis in zebrafish. Science *308*, 833–838.
18. Giraldez, A.J., Mishima, Y., Rihel, J., Grocock, R.J., Van Dongen, S., Inoue, K., Enright, A.J., and Schier, A.F. (2006). Zebrafish MiR-430 promotes deadenylation and clearance of maternal mRNAs. Science *312*, 75–79.
19. Fromm, B., Domanska, D., Høye, E., Ovchinnikov, V., Kang, W., Aparicio-Puerta, E., Johansen, M., Flatmark, K., Mathelier, A., Hovig, E., et al. (2020). MirGeneDB 2.0: the metazoan microRNA complement. Nucleic Acids Res. *48* (D1), D132–D141.
20. Zhang, L., Ward, J.D., Cheng, Z., and Dernburg, A.F. (2015). The auxin-inducible degradation (AID) system enables versatile conditional protein depletion in C. elegans. Development *142*, 4374–4384.
21. Drake, M., Furuta, T., Suen, K.M., Gonzalez, G., Liu, B., Kalia, A., Ladbury, J.E., Fire, A.Z., Skeath, J.B., and Arur, S. (2014). A requirement for ERK-dependent Dicer phosphorylation in coordinating oocyte-to-embryo transition in C. elegans. Dev. Cell *31*, 614–628.
22. Minogue, A.L., Tackett, M.R., Atabakhsh, E., Tejada, G., and Arur, S. (2018). Functional genomic analysis identifies miRNA repertoire regulating C. elegans oocyte development. Nat. Commun. *9*, 5318.
23. McJunkin, K., and Ambros, V. (2014). The embryonic *mir-35* family of microRNAs promotes multiple aspects of fecundity in *Caenorhabditis elegans*. G3 *4*, 1747–1754.
24. Lehrbach, N.J., Castro, C., Murfitt, K.J., Abreu-Goodger, C., Griffin, J.L., and Miska, E.A. (2012). Post-developmental microRNA expression is required for normal physiology, and regulates aging in parallel to insulin/IGF-1 signaling in C. elegans. RNA *18*, 2220–2235.
25. Miska, E.A., Alvarez-Saavedra, E., Abbott, A.L., Lau, N.C., Hellman, A.B., McGonagle, S.M., Bartel, D.P., Ambros, V.R., and Horvitz, H.R. (2007). Most Caenorhabditis elegans microRNAs are individually not essential for development or viability. PLoS Genet. *3*, e215.
26. Alvarez-Saavedra, E., and Horvitz, H.R. (2010). Many families of C. elegans microRNAs are not essential for development or viability. Curr. Biol. *20*, 367–373.
27. Shaw, W.R., Armisen, J., Lehrbach, N.J., and Miska, E.A. (2010). The conserved miR-51 microRNA family is redundantly required for embryonic development and pharynx attachment in Caenorhabditis elegans. Genetics *185*, 897–905.
28. Alberti, C., Manzenreither, R.A., Sowemimo, I., Burkard, T.R., Wang, J., Mahofsky, K., Ameres, S.L., and Cochella, L. (2018). Cell-type specific sequencing of microRNAs from complex animal tissues. Nat. Methods *15*, 283–289.

CellPress
OPEN ACCESS

29. Martinez, N.J., Ow, M.C., Reece-Hoyes, J.S., Barrasa, M.I., Ambros, V.R., and Walhout, A.J.M. (2008). Genome-scale spatiotemporal analysis of Caenorhabditis elegans microRNA promoter activity. Genome Res. *18*, 2005–2015.

30. Okamura, K., Hagen, J.W., Duan, H., Tyler, D.M., and Lai, E.C. (2007). The mirtron pathway generates microRNA-class regulatory RNAs in Drosophila. Cell *130*, 89–100.

31. Ruby, J.G., Jan, C.H., and Bartel, D.P. (2007). Intronic microRNA precursors that bypass Drosha processing. Nature *448*, 83–86.

32. Massirer, K.B., Perez, S.G., Mondol, V., and Pasquinelli, A.E. (2012). The miR-35-41 family of microRNAs regulates RNAi sensitivity in Caenorhabditis elegans. PLoS Genet. *8*, e1002536.

33. Bartel, D.P. (2009). MicroRNAs: target recognition and regulatory functions. Cell *136*, 215–233.

34. Reimão-Pinto, M.M., Ignatova, V., Burkard, T.R., Hung, J.-H., Manzenreither, R.A., Sowemimo, I., Herzog, V.A., Reichholf, B., Fariña-Lopez, S., and Ameres, S.L. (2015). Uridylation of RNA hairpins by tailor confines the emergence of microRNAs in Drosophila. Mol. Cell *59*, 203–216.

35. Bortolamiol-Becet, D., Hu, F., Jee, D., Wen, J., Okamura, K., Lin, C.-J., Ameres, S.L., and Lai, E.C. (2015). Selective suppression of the splicing-mediated microRNA pathway by the terminal uridyltransferase tailor. Mol. Cell *59*, 217–228.

36. Lee, R.C., Feinbaum, R.L., and Ambros, V. (1993). The C. elegans heterochronic gene lin-4 encodes small RNAs with antisense complementarity to lin-14. Cell *75*, 843–854.

37. Reinhart, B.J., Slack, F.J., Basson, M., Pasquinelli, A.E., Bettinger, J.C., Rougvie, A.E., Horvitz, H.R., and Ruvkun, G. (2000). The 21-nucleotide let-7 RNA regulates developmental timing in Caenorhabditis elegans. Nature *403*, 901–906.

38. Johnston, R.J., Jr., and Hobert, O. (2003). A microRNA controlling left/right neuronal asymmetry in Caenorhabditis elegans. Nature *426*, 845–849.

39. Grimson, A., Srivastava, M., Fahey, B., Woodcroft, B.J., Chiang, H.R., King, N., Degnan, B.M., Rokhsar, D.S., and Bartel, D.P. (2008). Early origins and evolution of microRNAs and Piwi-interacting RNAs in animals. Nature *455*, 1193–1197.

40. McJunkin, K., and Ambros, V. (2017). A microRNA family exerts maternal control on sex determination in *C. elegans*. Genes Dev. *31*, 422–437.

41. Sherrard, R., Luehr, S., Holzkamp, H., McJunkin, K., Memar, N., and Conradt, B. (2017). miRNAs cooperate in apoptosis regulation during *C. elegans* development. Genes Dev. *31*, 209–222.

42. Tran, A.T., Chapman, E.M., Flamand, M.N., Yu, B., Krempel, S.J., Duchaine, T.F., Eroglu, M., and Derry, W.B. (2019). MiR-35 buffers apoptosis thresholds in the C. elegans germline by antagonizing both MAPK and core apoptosis pathways. Cell Death Differ. *26*, 2637–2651.

43. Doll, M.A., Soltanmohammadi, N., and Schumacher, B. (2019). ALG-2/AGO-dependent *mir-35* family regulates DNA damage-induced apoptosis through MPK-1/ERK MAPK signaling downstream of the core apoptotic machinery in *Caenorhabditis elegans*. Genetics *213*, 173–194.

44. Boeck, M.E., Huynh, C., Gevirtzman, L., Thompson, O.A., Wang, G., Kasper, D.M., Reinke, V., Hillier, L.W., and Waterston, R.H. (2016). The time-resolved transcriptome of C. elegans. Genome Res. *26*, 1441–1450.

45. Parchem, R.J., Moore, N., Fish, J.L., Parchem, J.G., Braga, T.T., Shenoy, A., Oldham, M.C., Rubenstein, J.L., Schneider, R.A., and Blelloch, R. (2015). miR-302 is required for timing of neural differentiation, neural tube closure, and embryonic viability. Cell Rep. *12*, 760–773.

46. Drexel, T., Mahofsky, K., Latham, R., Zimmer, M., and Cochella, L. (2016). Neuron type-specific miRNA represses two broadly expressed genes to modulate an avoidance behavior in C. elegans. Genes Dev. *30*, 2042–2047.

47. Wienholds, E., Kloosterman, W.P., Miska, E., Alvarez-Saavedra, E., Berezikov, E., de Bruijn, E., Horvitz, H.R., Kauppinen, S., and Plasterk, R.H. (2005). MicroRNA expression in zebrafish embryonic development. Science *309*, 310–311.

48. Rahman, R.-U., Liebhoff, A.-M., Bansal, V., Fiosins, M., Rajput, A., Sattar, A., Magruder, D.S., Madan, S., Sun, T., Gautam, A., et al. (2020). SEAweb: the small RNA Expression Atlas web application. Nucleic Acids Res. *48* (D1), D204–D219.

49. Heidersbach, A., Saxby, C., Carver-Moore, K., Huang, Y., Ang, Y.-S., de Jong, P.J., Ivey, K.N., and Srivastava, D. (2013). MicroRNA-1 regulates sarcomere formation and suppresses smooth muscle gene expression in the mammalian heart. eLife *2*, e01323.

50. Simon, D.J., Madison, J.M., Conery, A.L., Thompson-Peer, K.L., Soskis, M., Ruvkun, G.B., Kaplan, J.M., and Kim, J.K. (2008). The microRNA miR-1 regulates a MEF-2-dependent retrograde signal at neuromuscular junctions. Cell *133*, 903–915.

51. Nehammer, C., Ejlerskov, P., Gopal, S., Handley, A., Ng, L., Moreira, P., Lee, H., Issazadeh-Navikas, S., Rubinsztein, D.C., and Pocock, R. (2019). Interferon-β-induced miR-1 alleviates toxic protein accumulation by controlling autophagy. eLife *8*, 287.

52. Gutiérrez-Pérez, P., Santillán, E.M., Lendl, T., Schrempf, A., Steinacker, T.L., Asparuhova, M., Brandstetter, M., Haselbach, D., and Cochella, L. (2020). A deeply conserved miR-1 dependent regulon supports muscle cell physiology. bioRxiv. https://doi.org/10.1101/2020.08.31.275644.

53. Markham, N.R., and Zuker, M. (2005). DINAMelt web server for nucleic acid melting prediction. Nucleic Acids Res. *33*, W577–W581.

54. Di Tommaso, P., Chatzou, M., Floden, E.W., Barja, P.P., Palumbo, E., and Notredame, C. (2017). Nextflow enables reproducible computational workflows. Nat. Biotechnol. *35*, 316–319.

55. Brenner, S. (1974). The genetics of Caenorhabditis elegans. Genetics *77*, 71–94.

56. Chung, W.J., Agius, P., Westholm, J.O., Chen, M., Okamura, K., Robine, N., Leslie, C.S., and Lai, E.C. (2011). Computational and experimental identification of mirtrons in Drosophila melanogaster and Caenorhabditis elegans. Genome Res. *21*, 286–300.

57. Frank, F., Sonenberg, N., and Nagar, B. (2010). Structural basis for 5′-nucleotide base-specific recognition of guide RNA by human AGO2. Nature *465*, 818–822.

58. Zhang, H., and Blumenthal, T. (1996). Functional analysis of an intron 3′ splice site in Caenorhabditis elegans. RNA *2*, 380–388.

59. Paix, A., Folkmann, A., Rasoloson, D., and Seydoux, G. (2015). High efficiency, homology-directed genome editing in Caenorhabditis elegans using CRISPR-Cas9 ribonucleoprotein complexes. Genetics *201*, 47–54.

60. Prior, H., Jawad, A.K., MacConnachie, L., and Beg, A.A. (2017). Highly efficient, rapid and Co-CRISPR-independent genome editing in *Caenorhabditis elegans*. G3 (Bethesda) *7*, 3693–3698.

61. Lutzmayer, S., Enugutti, B., and Nodine, M.D. (2017). Novel small RNA spike-in oligonucleotides enable absolute normalization of small RNA-seq data. Sci. Rep. *7*, 5913.

62. Love, M.I., Huber, W., and Anders, S. (2014). Moderated estimation of fold change and dispersion for RNA-seq data with DESeq2. Genome Biol. *15*, 550.

STAR★METHODS

KEY RESOURCES TABLE

REAGENT or RESOURCE	SOURCE	IDENTIFIER
Antibodies		
Mouse monoclonal anti-myc (clone 4A6)	Merck Millipore	Cat# 05-724; RRID: AB_11211891
Rabbit polyclonal anti-gamma-tubulin	Abcam	Cat# ab50721; RRID: AB_880628)
Anti-mouse IgG secondary HRP-linked	Cell Signaling Technologies	Cat# 7076; RRID: AB_330924)
Anti-rabbit IgG secondary HRP-linked	Cell Signaling Technologies	Cat# 7074; RRID: AB_2099233)
Bacterial and Virus Strains		
E. coli OP50 standard *C. elegans* food source	CGC	N/A
E. coli HT115 RNAi bacteria (targeting pash-1)	ORFeome version 1.1	T22A3.5 (ORF-ID)
Chemicals, Peptides, and Recombinant Proteins		
3-Indolacetic acid (Auxin)	Sigma-Aldrich	CAS I2886
Cas-9 from *Staphylococcus aureus*	In-house	N/A
Critical Commercial Assays		
TRIzol Reagent	Invitrogen	CAS 15596026
SuperScript III	Invitrogen	CAS 18080044
GoTAQ qPCR Mastermix	Promega	CAS A6001
Alt-R CRISPR-Cas9 tracr/crRNA system for genome editing (insertion of AID-tags)	Integrated DNA technologies	N/A
Deposited Data		
Small RNAseq data deposition with NCBI GEO	This paper	GEO: GSE153233
Experimental Models: Organisms/Strains		
C. elegans strains used/generated: see Table S1	CGC / this paper	N/A
Oligonucleotides		
qPCR-primer: pash-1-F: 5′-GCCTTCGAGAAAACGGGGAA-3′	This paper	N/A
qPCR-primer: pash-1-R: 5′-TGGCTCCCATTTCGGAGATT-3′	This paper	N/A
qPCR-primer: drsh-1-F: 5′-TGAGCTGGCTTTGGCTAATCT-3′	This paper	N/A
qPCR-primer: drsh-1-R: 5′-ACCCCGTAATTAGTCGCTGG-3′	This paper	N/A
qPCR-primer: cdc-42-F: 5′-TGGGTGCCTGAAATTTCGC-3′	This paper	N/A

(*Continued on next page*)

Continued

REAGENT or RESOURCE	SOURCE	IDENTIFIER
qPCR-primer: cdc-42-R: 5′-CTTCTCCTGTTGTGG TGGG-3′	This paper	NA
ALT-R crRNA drsh-1 N terminus: 5′-TTAGATTTTCATTT AGATGT-3′	This paper	N/A
ALT-R crRNA drsh-1 C terminus: 5′-GATACCAGCGACT AATTACG-3′	This paper	N/A
ALT-R crRNA pash-1 C terminus: 5′-AGGTGAATATACT ATTTGTG-3′	This paper	N/A
Recombinant DNA		
Plasmid pPD75_95 (base vector for mirtrons)	Fire Lab / Addgene	CAS 1494
Expression vectors for mirtron-35 and mirtron-51 (see STAR Methods or Figure S2 for details)	This paper	N/A
Software and Algorithms		
RNAfold Two-State-Folding algorithm	[53]	http://unafold.rna.albany.edu/
miRNA analysis pipeline for sRNaseq data	[28]	https://doi.org/10.1038/nmeth.4610
Nextflow pipeline	[54]	https://www.nextflow.io/about-us.html
Nextflow pipeline modifications	This paper	https://github.com/lengfei5/smallRNA_nf/tree/master/dev_sRBC
Scripts for analysis of sRNaseq data presented here	This paper	https://github.com/lengfei5/smallRNA_analysis_philipp/tree/master/scripts

RESOURCE AVAILABILITY

Lead Contact

Further information and requests for resources and reagents should be directed to and will be fulfilled by the Lead Contact, Luisa Cochella (cochella@imp.ac.at).

Materials Availability

Key strains will be made available at the Caenorhabditis Genetics Center (University of Minnesota), other strains and plasmids are available upon request.

Data and Code Availability

Sequencing data are deposited under the GEO accession number GEO: GSE153233. Small RNA reads were mapped and processed as in [28]. Modifications of the Nextflow pipeline for can be found under: https://github.com/lengfei5/smallRNA_nf/tree/master/dev_sRBC. Other scripts can be found at: https://github.com/lengfei5/smallRNA_analysis_philipp/tree/master/scripts

EXPERIMENTAL MODEL AND SUBJECT DETAILS

Caenorhabditis elegans was kept under standard conditions on NGM plates seeded with *Escherichia coli* OP50 at 20°C as previously described [55], unless indicated otherwise. The Bristol strain N2 was used as a wild-type control. MiR-35 family mutant embryos were

derived from self-fertilized, homozygous mutant mothers (nDf49, nDf50) that were maternally rescued. For analysis of the earliest miR-51 family mutant phenotypes, we sought to reduce the maternal load of miR-51 family miRNAs (as embryos can only be obtained from mothers expressing at least one mir-51 family member). To this end, mutant embryos were derived from mothers expressing miR-54-56 as well as an early embryonic mir-35^{prom}::GFP reporter from an extrachromosomal array, while all genomic copies of miR-51 family miRNAs were deleted (nDf58, nDf67, n4100). These arrays tend to be lowly expressed or silenced in the *C. elegans* germline and are only segregated to a fraction of the offspring. Therefore, miR-51 family mutant embryos obtained from these mothers upon self-fertilization were selected for lack of the rescuing array by absence of mir-35^{prom}::GFP expression, and subsequently analyzed. A full list of strains used and generated in this study is provided in Table S1.

METHOD DETAILS

Mirtron design and expression

Production of a miRNA of interest via the mirtron pathway involves the challenge of designing an intron that resembles a pre-miRNA hairpin and simultaneously allows for efficient splicing. To generate mirtron-versions of miR-35 or miR-51, the 6 nucleotides at the 3′ end of the respective pre-miRNA-hairpin were modified to create a 3′ splice site and match the consensus sequence of 12 annotated endogenous *C. elegans* mirtrons (Figure S2A) [56]. Because intron borders correspond to the ends of mature miRNAs, the 5′ arm of mirtrons invariably starts with a G, which negatively impacts loading into Argonaute [57]. Thus, mature mirtrons are preferably incorporated into the 3′ arm. As interaction of miRNAs with cognate target mRNAs is largely determined by the seed region (nts 2-8), minor alterations in the 3′ end of a miRNA are expected to have little impact on its target repertoire. As branch point motifs are rather permissive in *C. elegans*, virtually any A located in vicinity to a 3′ splice site allows for efficient lariat formation [58], we thus made no additional changes to account for the branch point. Upon introduction of a functional 3′ splice site into the 3p-arm of the pre-miRNA, compensatory changes were introduced in the 5p arm to retain hairpin secondary structure. This was guided by secondary structure predictions using the RNAfold Two-State-Folding algorithm for RNA at 20°C [53], yielding a mirtron-hairpin with a secondary structure highly similar to its endogenous pre-miRNA-hairpin equivalent (Figure S2C). The resulting sequence was used to replace the middle intron of GFP in the commonly employed *C. elegans* vector pPD75.95 (Fire lab). To recapitulate the spatio-temporal expression pattern of endogenous miRNAs, the promoter sequence of the respective endogenous miRNA was used to drive mirtron expression (2.0 kb upstream of pre-mir-35 or 2.5 kb upstream of pre-mir-52, respectively). Conveniently, GFP-fluorescence serves as an internal control for correct splicing of the inserted mirtron. Functionality of mirtrons was assayed by testing their ability to rescue an otherwise lethal deletion of the respective miRNA family. For the design of mirtron-35$_{\text{seed mutant}}$, three base pairs in the seed region of mirtron-35 were swapped in the pre-mirtron stem-loop, preserving integrity and thermodynamic features of the hairpin and miRNA-duplex to ensure efficient processing and loading of the mirtron-variant.

Genome engineering

To generate AID-tagged copies of *pash-1* and *drsh-1,* animals were subjected to Cas-9 mediated genome engineering via RNP-microinjection following the Co-CRISPR approach described by [59], with modifications from [60]. Briefly, the Alt-R CRISPR/Cas9 system (Integrated DNA Technologies) was employed and animals were injected with a mixture containing 300mM KCl, 20mM HEPES, 4μg/μl recombinant Cas9 (from *S. aureus,* purified in-house), 500ng/μl TracerRNA, 100ng/μl crRNA targeting the Co-CRISPR marker gene dpy-10, and 15ng/μl dpy-10- > rol-6 repair template oligo. Additionally, 100 ng/μl crRNA against the locus of interest as well as 100-200ng/μl dsDNA repair template encoding the desired modification with around 50bp flanking homology arms were added to the mix. Note that for efficient degradation of DRSH-1 we needed to insert AID degrons at the N- and C-termini simultaneously as either degron alone was insufficient for depletion. Guide RNAs used and sequences inserted (in bold) were as follows:

drsh-1 – N-terminal AID-tag – guide sequence used = 5′- TTAGATTTTCATTTAGATGT-3′ – locus post-edit:
TAGTTAACGTTTTCATCTGAAATTGTAGACAGATTTAGATTTTCATTTAG**ATGGAACAGAAACTCATCTCTGAAGAGGATCTG*AT***
GCCTAAAGATCCAGCCAAACCTCCGGCCAAGGCACAAGTTGTGGGATGGCCACCGGTGAGATCATACCGGAAGAACGTG
***ATGGTTTCCTGCCAAAAATCAAGCGGTGGCCCGGAGGCGGCGGCGTTCGTGAAG*GACTACAAAGACCATGACGGTGATT**
ATAAAGATCATGACATCGACTACAAGGATGACGATGACAAGGGAGGAAGCGGAGGAGGAAGCGGTGGAGGAAGCGGAGG
AGGAAGCGGAATGTCGGACGAAAAGATTTCAATGACGCTTAACTTCCCGAAACACAAGCG
drsh-1 – C-terminal AID-tag - guide sequence used = 5′-GATACCAGCGACTAATTACG-3′ – locus post-edit:
TCAAGTGGTTTCAGAACATGCGCCGTCGTCTTGAACAAGATACCAGCGACGGAGGCAGCGGTGGTGGAAGCGGCGGGGGAAG
CGGCGGTGGAAGCGGTGACTACAAAGACCATGACGGTGATTATAAAGATCATGACATCGACTACAAGGATGACGATGACAAG*A*
*TGCCTAAAGATCCAGCCAAACCTCCGGCCAAGGCACAAGTTGTGGGATGGCCACCGGTGAGATCATACCGGAAGAACGT****GA***
***TGGTTTCCTGCCAAAAATCAAGCGGTGGCCCGGAGGCGGCGGCGTTCGTGAAG*GAACAGAAACTCATCTCTGAAGAGGA**
TCTGTAGTTACGGGGTTATAATTATACTATGTCTGTTTGAATGTGATTCGGTTC
pash-1 – C-terminal AID-tag – guide sequence used = 5′-AGGTGAATATACTATTTGTG-3′ – locus post-edit:
GGGGAAGACATGACGATTCATCATCCCCATCACATCAGAAACCACACAAA**GGAGGAAGCGGAGGAGGAAGCGGAGACTACA**
AAGACCATGACGGTGATTATAAAGATCATGACATCGACTACAAGGATGACGATGACAAG*ATGCCTAAAGATCCAGCCAAA*
CCTCCGGCCAAGGCACAAGTTGTGGGATGGCCACCGGTGAGATCATACCGGAAGAACGTGATGGTTTCCTGCCAAAAATC

AAGCGGTGGCCCGGAGGCGGCGGCGTTCGTGAAG**GAACAGAAACTCATCTCTGAAGAGGATCTGTAG**TATATTCACCTCAT ATGTTTGTTGTTTTGTTGGTAGTTTTAATTTTT

Inserted sequences contained the TIR-1 recognition peptide (italics), a FLAG (GACTACAAAGACCATGACGGTGATTATAAAGAT CATGACATCGACTACAAGGATGACGATGACAAG) and a MYC (GAACAGAAACTCATCTCTGAAGAGGATCTG) tag, as well as GGSG linkers in between.

Pash-1(ts) experiments

Animals bearing the *pash-1(ts)* allele *mj100* were kept constantly at the permissive temperature of 16°C. *pash-1(ts)* animals expressing the mirtron-51 transgene were selected every few generations for high levels of the co-expression marker *elt-2p::dsRed*, as this transgene was prone to undergo silencing. For experiments, L4 stage larvae were transferred to a fresh plate and shifted to 25°C approximately 16h later. In order to achieve maximal depletion of maternal miRNAs, mothers were kept at the restrictive temperature an additional 7h before harvesting early embryos (1-4 cell stage). Extending the time at the restrictive temperature was prohibited by the rapidly deteriorating health of gravid *pash-1(ts)* adults expressing mirtrons at 25°C. For backshift-experiments with mirtron-rescued *pash-1(ts)* animals, hatched L1s that developed under restrictive conditions were transferred to a fresh plate within 1h after hatching, allowed to develop at 16°C, and scored for their ability to reach adulthood within 7 days.

RNAiD experiments

Animals were kept constantly on standard NGM plates seeded with *E. coli* OP50 at 20°C. For combined AID and RNAi treatment, NGM plates were supplemented with 1mM Carbenicillin, 1mM IPTG and 4mM Auxin (3-Indoleacetic acid, Sigma #I2886). RNAiD plates were seeded with *E. coli* HT115 expressing dsRNA which elicits an RNAi response against *pash-1* mRNA (clone from the ORFeome-RNAi v1.1 (Vidal) library, ORF-ID T22A3.5). To ensure efficient mRNA knockdown, RNAi bacteria were grown freshly for every experiment in liquid LB culture, the medium was brought to 1mM IPTG around 1h before pelleting bacteria, and plates were seeded with a 20X concentrate (as Auxin inhibits bacterial growth). For experiments, L4s were transferred to RNAiD plates, embryos were harvested 24h later, and assayed as described below. While the AID-system rapidly depletes proteins within minutes to hours [20], extending the Auxin treatment to 24h was required to ensure near-complete elimination of maternal miRNAs. Surprisingly, overall health of mirtron-expressing gravid adults upon RNAiD treatment was significantly less adversely affected compared to *pash-1(ts)* animals, eventually owed to the temperature difference and/or deleterious side-effects of PASH-1(TS) at 25°C. Efficiency of RNAi-mediated *pash-1* mRNA knockdown was assessed via RT-qPCR. Briefly, 5-10 embryos in the 2-cell stage were transferred to about 1 µl of Lysis Buffer (5mM Tris-HCl pH 8.0, 0.25 mM EDTA and 1 mg/mL Proteinase K, 0.5% Triton X-100, 0.5% Tween20) using a thin glass needle. These samples were subjected to 10 min digestion at 65°C before heat inactivation of proteinase K for 1 min at 85°C. Next, crude lysates were reverse transcribed before performing qPCR using the GoTAQ qPCR Mastermix (Promega) according to the manufacturer's instructions. Relative expression was calculated according to the $\Delta\Delta$Cq-method using *cdc-42* as a reference gene, the following primer sequences were employed:

pash-1-F: 5′-GCCTTCGAGAAAACGGGGAA-3′; *pash-1*-R: 5′-TGGCTCCCATTTCGGAGATT-3′;
drsh-1-F: 5′-TGAGCTGGCTTTGGCTAATCT-3′; *drsh-1*-R: 5′-ACCCCGTAATTAGTCGCTGG-3′
cdc-42-F: 5′-TGGGTGCCTGAAATTTCGC-3′; *cdc-42*-R: 5′-CTTCTCCTGTTGTGGTGGG-3′

Western blotting

To assess the extent of protein degradation of PASH-1:AID:MYC and DRSH-1:AID:MYC upon Auxin treatment, L4s were transferred to Auxin plates prepared as described above. 24h later, gravid adults were collected in Lämmli-buffer containing 2.5% β-Mercaptoethanol (v/v), and subjected to multiple cycles of snap-freezing followed by boiling until embryos were disrupted. Proteins were separated via SDS-PAGE and transferred onto a Nitrocellulose membrane. As primary antibodies either monoclonal mouse anti-myc (clone 46A, Merck Millipore, catalog number 05-724, diluted 1:2000) or a polyclonal rabbit anti-gamma tubulin (Abcam, catalog number ab50721, diluted 1:1,000) were used. As secondary antibody, anti-mouse IgG HRP-linked Antibody (Cell Signaling Technology, #7076, diluted 1:2,000) or anti-rabbit IgG HRP-linked Antibody (Cell Signaling Technology, #7074, dilution of 1:2,000) was applied followed by visualization using ECL reagent (Thermo Scientific).

Hatching assays

Embryos were obtained from day 1 gravid adults reared as described above, by slicing mothers in a drop of M9 buffer on a microscopic slide. 2-cell embryos of normal size were transferred to standard NGM plates or RNAiD plates and allowed to develop at the respective temperatures. Hatched animals were collected for subsequent analysis (backshift experiments, microscopy, or small RNA sequencing) from plates within 1h after hatching. The final hatching rate was assessed > 24h after embryo collection by scoring the fraction of animals that successfully escaped the eggshell. To obtain samples at specific developmental stages for small RNA sequencing, 2-cell embryos were harvested via slicing, collected or allowed to develop on NGM plates before being selected manually by stage at given time points (gastrulation: 4-5h after 2-cell stage, 3-fold = 8-9 hours after 3-cell stage).

Microscopy

For phenotypic analysis of embryonic arrest or hatched L1 larvae, animals were mounted on a thin agar-pad on a microscopic slide sealed with a coverslip. Images were recorded at 400x magnification using an AxioImager Z2 (Zeiss) equipped with DIC and fluorescence optics, and analyzed via ImageJ. Arrest phenotypes were scored as follows: cell mass = embryo fails to initiate bean stage; morphogenesis = embryo begins to elongate but fails to reach 2-fold stage; elongation = embryo completes 2-fold stage but arrests before hatching. Body measurements are based on DIC micrographs. Body length was measured as the length of a segmented path running through the mid body axis from head to tail. Body width was assessed as the arithmetic mean of 3 independent width measurements in the anterior, middle, and posterior part of each animal. Pharynx length was determined by the length of a segmented path running through the pharynx middle axis in animals expressing *myo-2*^{prom}*::mCherry*. Mean and range are plotted for each genotype; unpaired t test was used for statistical comparison.

Small RNA sequencing

To profile miRNA levels, a modified version of the small RNA sequencing protocol described in [28] was performed. Samples containing each between 25 and 100 embryos (yielding about 5 to 20 ng total RNA) were collected at indicated stages and snap-frozen in liquid nitrogen. A series of eight RNA spike-In oligos spanning a 500-fold range of concentrations [61] were added on a per embryo basis, and total RNA was extracted using TRIzol Reagent (Invitrogen) according to the recommendations by the manufacturer for cell samples. To ensure complete disruption of embryos, samples were snap-frozen and thawed multiple times before proceeding with the extraction. After total RNA purification samples were treated as described in [28], with two important changes: A) amounts of 3′ linker and 5′ linker were reduced to a final concentration of 500 nM to reduce undesired amplification products. B) barcodes and random nucleotides serving as unique molecular identifiers were introduced into the 3′ linker to circumvent low-input related contamination and over-amplification issues, resulting in ligation products of the following sequence:

(XXXXX = barcode): 5′-ACACUCUUUCCCUACACGACGCUCUUCCGAUCUNNNN-(sRNA)$_{18\text{-}30nt}$-NNNNNNXXXXXAGATCGGAAGAGCAC-ACGTCT/3ddC/-3′. After sufficient PCR-amplification as observed by SYBR green derived qPCR signal (requiring around 16-20 cycles), libraries were size-selected on an agarose gel to remove adaptor dimers and sequenced on a HiSeqV4 platform (Illumina). Small RNA reads were mapped and processed as in [28]. Modifications include addition of 3′ adaptor-barcode demultiplexing, which was added after trimming adaptor sequence in the Nextflow-based pipeline [54]. The modified Nextflow pipeline can be found at: https://github.com/lengfei5/smallRNA_nf/tree/master/dev_sRBC. To accurately quantify sRNAs, both mapped sequences and associated random nucleotides (functioning as UMIs) within the raw reads were quantified for miRNAs and piRNAs as well as spike-ins. To alleviate PCR amplification bias, UMI counts were used and normalized to spike-in as described in [61], allowing for absolute quantification of miRNA content per embryo even under conditions in which most miRNAs are depleted. In addition, as piRNAs are not affected across conditions and can thus be considered an internal control, data was normalized in parallel using total number of piRNA reads, yielding results highly similar to the ones obtained by spike-in normalization and providing additional confidence in the quantitative nature of the data. Scripts for this analysis can be found at: https://github.com/lengfei5/smallRNA_analysis_philipp/tree/master/scripts. Every round of library preparation included one sample of a comparable amount of *Arabidopsis thaliana* total RNA to assess the extent of potential contamination. The average counts for reads mapping to *C. elegans* miRNAs in contamination control samples (~1% of the wild-type miRNA content) across four independent library preparations were subtracted from all samples as a background correction. For background-corrected, spike-in normalized, miRNA counts in molecules per embryo, see Table S2. Raw data from sRNA-seq experiments has been deposited in the NCBI-GEO database under the accession number GEO: GSE153233.

QUANTIFICATION AND STATISTICAL ANALYSIS

Plots were created using Prism, statistical analysis was also performed in Prism as described in the figure legends. For sRNAseq data statistical analysis was performed using DESeq2 [62]. Figures were prepared with Adobe Illustrator CS6.

Current Biology, Volume *30*

Supplemental Information

Two MicroRNAs Are Sufficient for Embryonic Patterning in *C. elegans*

Philipp J. Dexheimer, Jingkui Wang, and Luisa Cochella

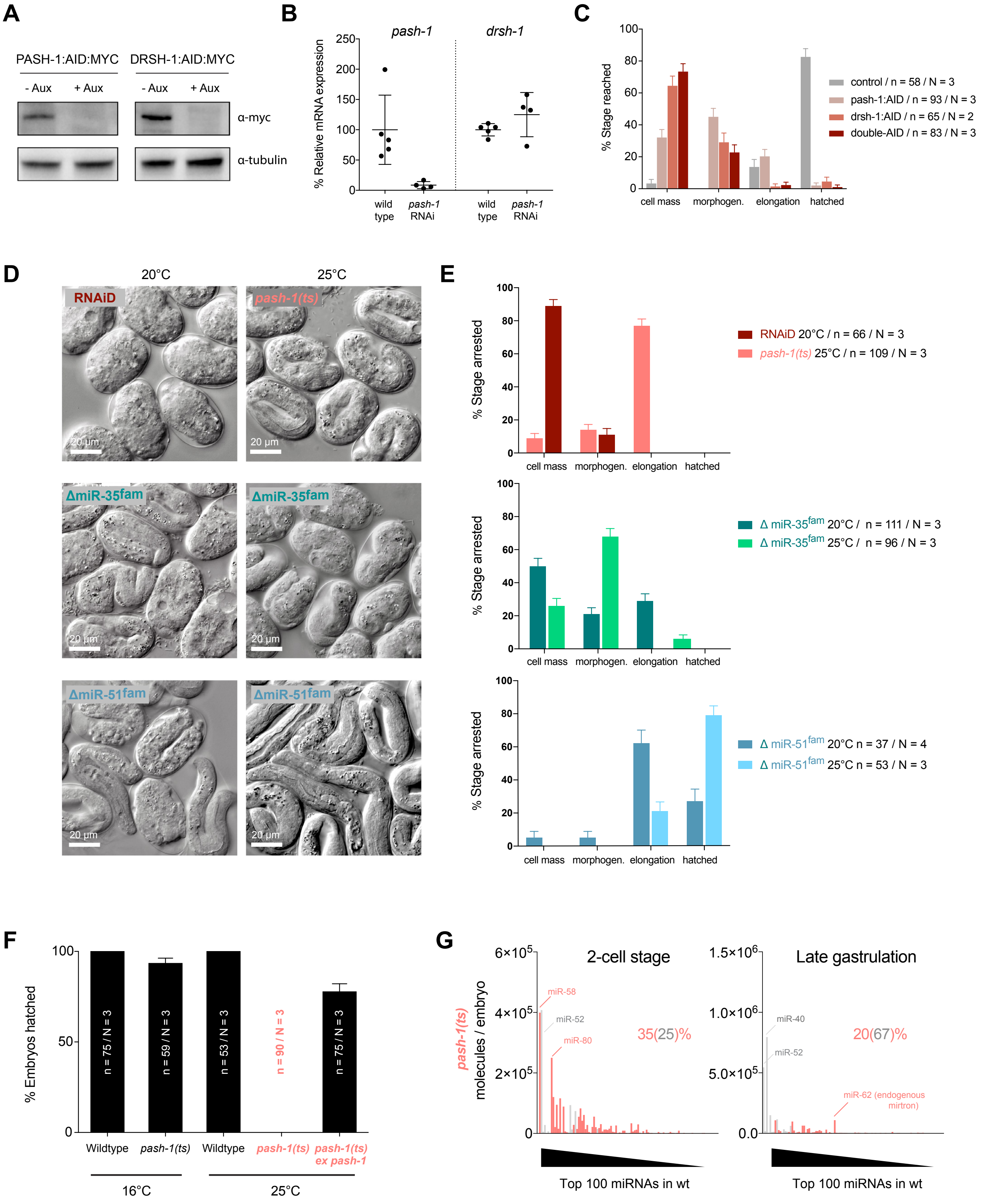

Figure S1. miRNAs are essential for morphogenesis and organogenesis. Related to Figure 1. A) Western Blot against endogenous, AID-Myc-tagged PASH-1 and DRSH-1 in gravid adults, 24h post Auxin treatment. **B**) mRNA levels measured by RT-qPCR in 2-cell embryos, 24h post pash-1 RNAi treatment compared to wild type under standard conditions. **C**) Quantification of the arrest phenotype of various strains bearing AID-tagged PASH-1 and/or DRSH-1 and expressing the TIR1 ubiquitin ligase, or TIR1 only as control, upon Auxin treatment. **D, E**) Images and quantification of arrest phenotype of RNAiD, *pash-1(ts)*, miR-35^{fam}, and miR-51^{fam} mutant embryos at 20°C and 25°C. **F**) Hatching assay of embryos bearing the pash-1(ts) temperature-sensitive allele mj100. Penetrant embryonic lethality at the restrictive temperature 25°C is rescued by expression of wild-type pash-1 from an extra-chromosomal array. **G**) miRNA-profile of pash-1(ts) embryos at the 2-cell stage and at the end of gastrulation as determined by small RNA sequencing using spike-in oligos for quantitative measure of absolute miRNA levels across samples, plotted as in Figure 1B. Note that the samples analyzed here are the same as in Figures S2G, 4A, S4A.

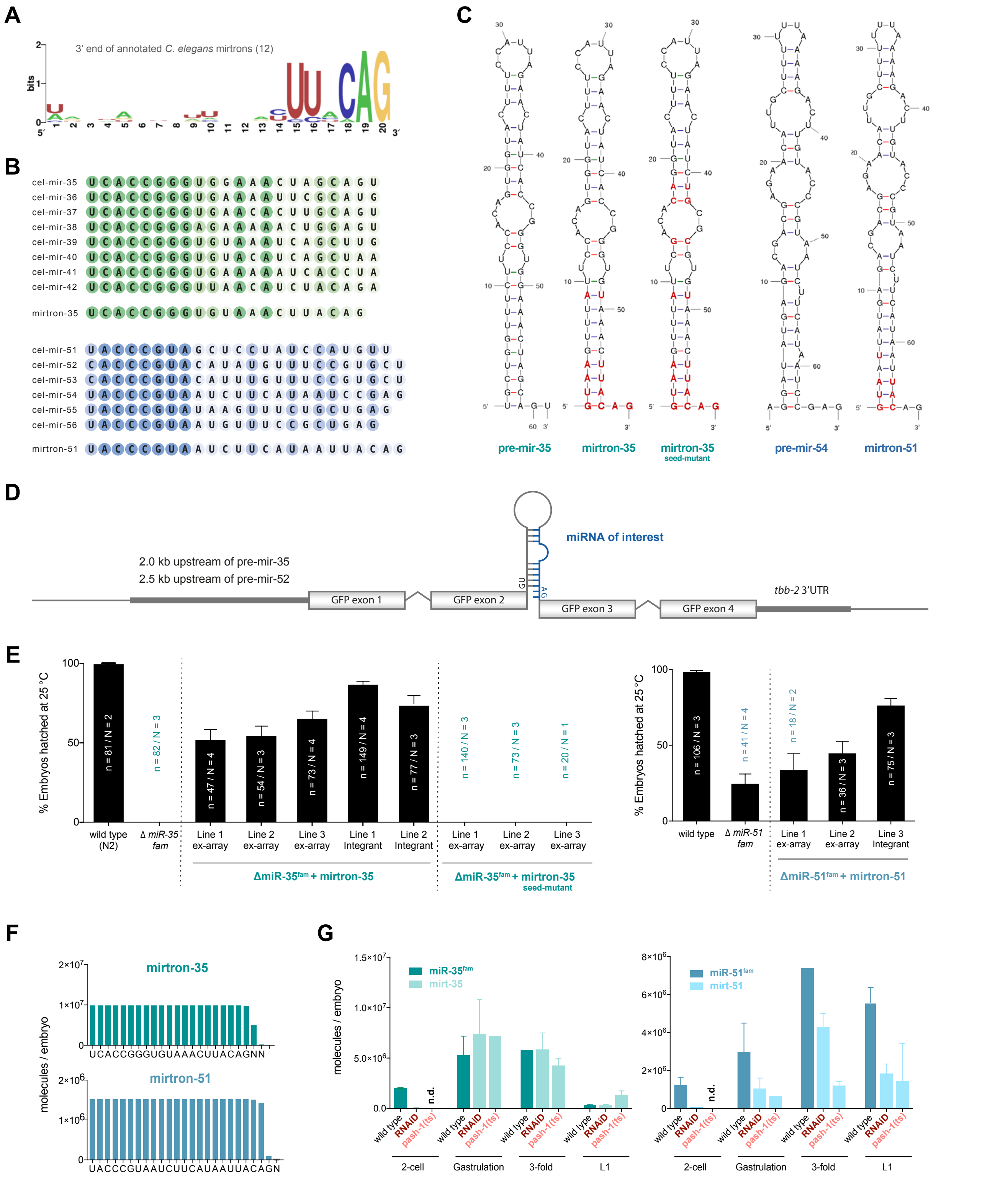

Figure S2. Mirtron design and expression. Related to Figure 2. A) Consensus sequence at the 3′ end of all 12 annotated *C. elegans* mirtrons (S1). **B**) Sequence of miR-35 and miR-51 family miRNAs and the corresponding designed mirtrons. **C**) Pre-miRNA hairpin structure for endogenous miRNA and mirtron counterpart as predicted by the UnaFold TwoState folding algorithm for RNA at 20ºC (S2); the basis for the design of mirtron-51 was the family member miR-54 as it required relatively few changes to be turned into a mirtron. Residues that have been changed in the mirtron hairpins are highlighted in red. **D**) Schematic design of mirtron-expressing transgenes. The endogenous promoter sequence upstream of *pre-mir-35* (2.0 kb) or *pre-mir-52* (2.5 kb) drives expression of a GFP transcript bearing the mirtron in its middle intron, followed by an inert *tbb-2* (tubulin) 3′ UTR. **E**) Hatching rates of embryos bearing deletions for the miR-35 family (*nDf49, nDf50*), or the miR-51 family (*nDf58, nDf67, n4100*), respectively. Shown are multiple independent transgenic lines expressing the respective mirtron from an extra-chromosomal array or the integrated transgenes that were used for subsequent experiments. **F**) Mirtron-read abundance per nucleotide derived from sRNA-seq data from mirtron-expressing RNAiD embryos at gastrulation (mirtron read-profiles were highly similar for other time points). Mirtron-35 had an untemplated single-nucleotide addition in 25-45% of the reads, which was a U in >90% of cases, consistent with observations in *Drosophila* (S3, S4). Mirtron-51 was trimmed by one nucleotide in 90-95% of cases. **G**) Comparison of the sum of the abundances of all endogenous miRNA family members in wild-type embryos (dark shades) and the respective mirtron abundance in Microprocessor depleted animals (light shades) throughout embryogenesis, derived from quantitative sRNA-seq analysis. Mirtrons are not detected at the 2-cell stage, consistent with commonly observed silencing of multicopy transgenes in the *C. elegans* germline. Thus, germline-related phenotypes such as the loss in fertility are not rescued by the mirtron transgenes.

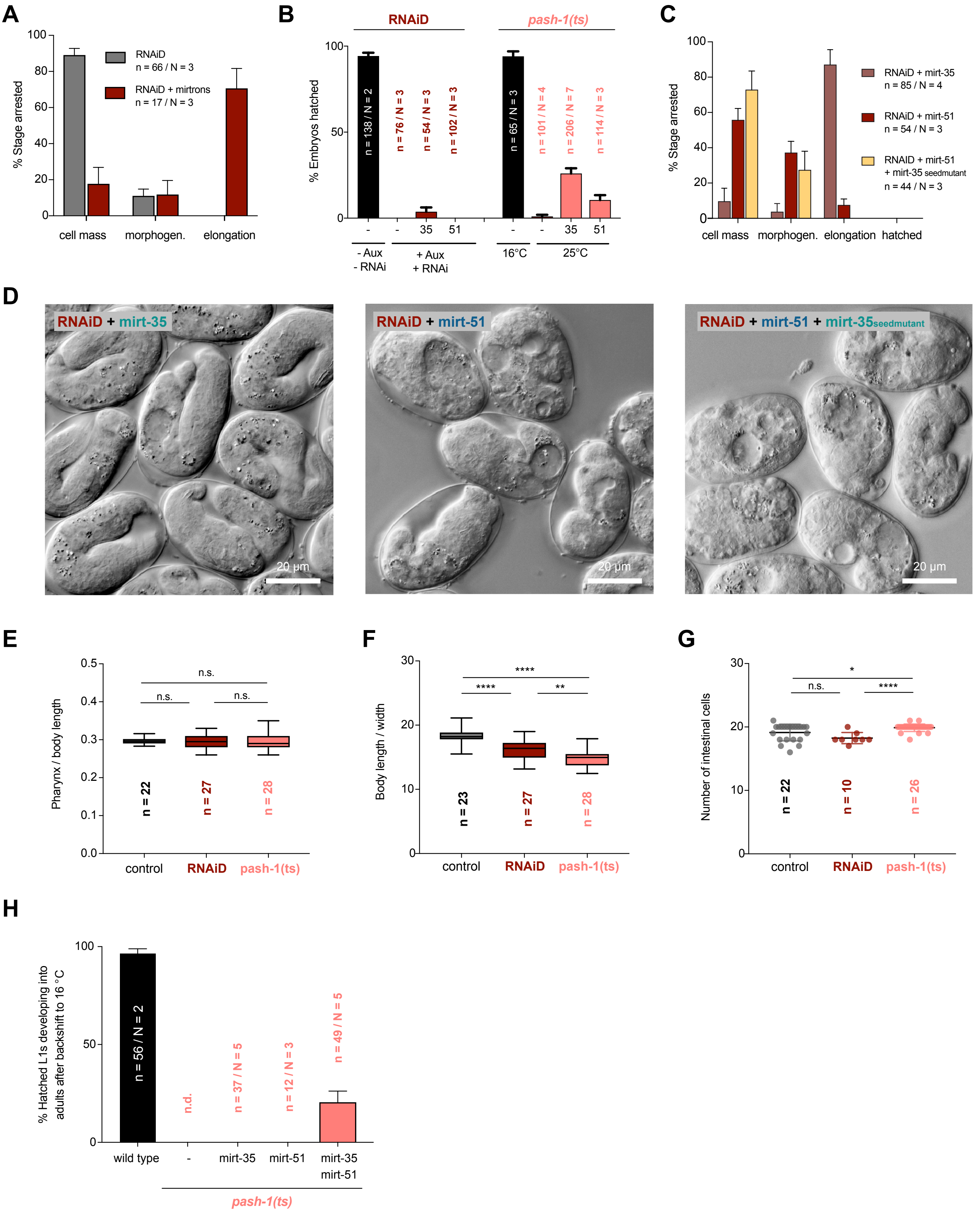

Figure S3. Two miRNAs are sufficient for embryonic patterning in the absence of Drosha and Pasha. Related to Figure 3. **A**) Stage of arrest in the ~50% of RNAiD embryos with mirtron-35 and -51 that fail to hatch, compared to embryos without mirtrons. For comparison data of RNAiD embyros is replotted from Figure S1E. **B**) Proportion of hatching of Microprocessor-deficient embryos expressing either mirtron-35 or mirtron-51 individually. **C**) Stage of arrest of RNAiD embryos expressing either mirtron individually, or mirtron-51 and the seed-mutant version of mirtron-35. For the seed-mutant mirt-35, three independent lines behaved identically of which one is shown. **D**) Representative images of animals scored in C. **E-G**) Body measurements of mirtron-rescued RNAiD or *pash-1(ts)* animals compared to wild type under standard conditions. Data is derived from micrographs using ImageJ. Pharynx length was measured using the expression area of a myo-2prom::mCherry reporter. Number of intestinal cells was scored by expression of a nuclear *elt-2prom::dsRed* reporter. Mean and range are plotted for each genotype; statistical significance was determined by an unpaired t-test, significance levels are: P>0.05 = n.s.; P<0.05 = *; P<0.01 = **; P<0.001 = ***. **H**) *pash-1(ts)* backshift experiment: miRNA-depleted embryos that developed at the restrictive temperature of 25°C and expressed mirt-35/51 individually or in combination, were transferred to the permissive temperature 16°C directly after hatching. Survival to adulthood was scored within the next 7 days.

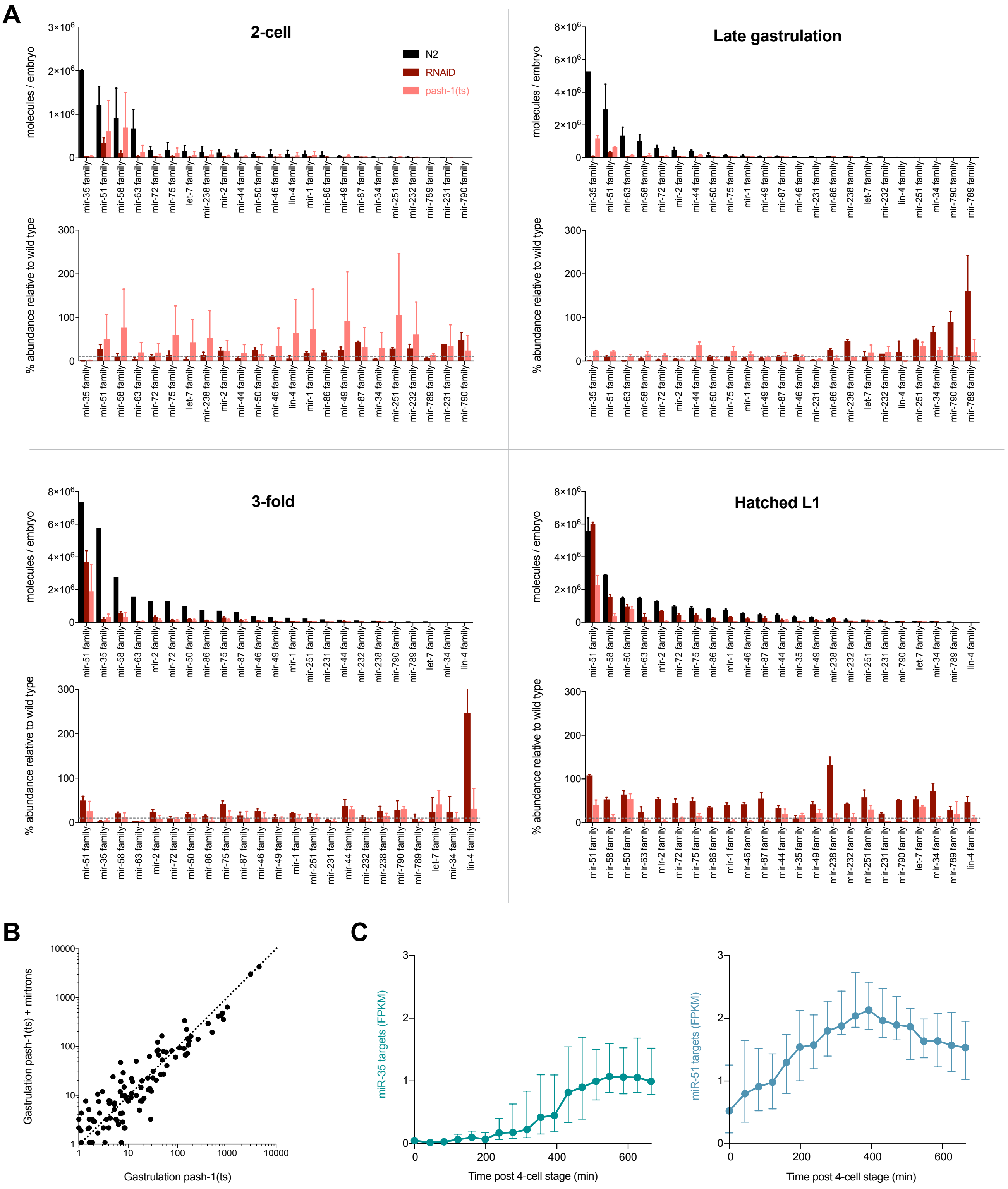

Figure S4. miRNA levels in microprocessor-depleted embryos. Related to Figure 4 and Table S2. A) For each timepoint profiled, shown are (top) the sum of the absolute abundances of canonical miRNAs grouped by families as measured by quantitative sRNA-seq (related to Figure 4A), and (bottom) the relative abundance of each miRNA family under RNAiD or pash-1(ts)-mediated depletion, in comparison to wild type. The horizontal dotted line indicates 10% of wild type. Depletion at the level of families is very strong except as noted also for individual miRNAs at the 2-cell stage in *pash-1(ts)* and at the L1 stage in RNAiD. There is no consistent leftover of other families over both depletion conditions that suggests that other miRNAs could contribute to early development. Note that the outliers in relative abundance at gastrulation and 3-fold stages correspond to families with very low absolute abundance. **B**) miRNA abundance at gastrulation in *pash-1(ts)* embryos with or without mirtrons. Mirtron-expression has no systematic effect on levels of left-over miRNAs. **C**) Embryonic expression time course of transcripts bearing conserved miR-35/51 family miRNA binding sites as predicted by TargetScan Worm 6.2 (S5, S6). Shown are median expression and 95% confidence intervals. For miR-35 family all 89 predicted targets are shown. For miR-51 family targets the top 71 expressing transcripts (out of 264 called targets in the dataset) are shown, selected based on an average FPKM value > 1 across all timepoints. The most abundant predicted miR-51^{fam} target *rps-20* (ribosomal protein, small subunit) has been removed as an outlier due to extreme abundance. Data derived from (S7).

Strain	Genotype	Comments	Figure
N2	-	wild type control strain (Brenner, 1974)	1B-D, S1B, S1F, 2B, S2E, S2G, 4A, S4A
**miR-35 family related**			
MT14533	nDf49, nDf50/mIn1 [mIs14 dpy-10(e128)] II	balanced mir-35 family deletion<br>(Alvarez-Saavedra and Horvitz, 2010)	1C, S1D, S1E, 2B, S2E
MLC812	nDf49, nDf50, II;<br>lucEx521 (mir35p::GFP_mirtron35::tbb2, myo2::mCherry)	mir-35 family deletion,<br>rescued by mirtron-35, line 1<br>generated by microinjection into MT14533	S2E
MLC825	nDf49, nDf50, II;<br>lucEx522 (mir35p::GFP_mirtron35::tbb2, myo2::mCherry)	mir-35 family deletion,<br>rescued by mirtron-35, line 2<br>generated by microinjection into MT14533	S2E
MLC826	nDf49, nDf50, II;<br>lucEx523 (mir35p::GFP_mirtron35::tbb2, myo2::mCherry)	mir-35 family deletion,<br>rescued by mirtron-35, line 3<br>generated by microinjection into MT14533	S2E
MLC1016	nDf49, nDf50, II;<br>lucIs20 (mir35p::GFP_mirtron35::tbb2, myo2::mCherry)	mir-35 family deletion,<br>rescued by mirtron-35, line 1<br>integrated via irradiation of MLC826	2B, S2E
MLC1017	nDf49, nDf50, II;<br>lucIs21 (mir35p::GFP_mirtron35::tbb2, myo2::mCherry)	mir-35 family deletion,<br>rescued by mirtron-35, line 2<br>integrated via irradiation of MLC826	S2E
MLC2353	nDf49, nDf50 / mIn1, II<br>lucEx1240 (mir35p::GFP_mirtron35(seed-mutant)::tbb2, myo2::mCherry)	balanced mir-35 family deletion,<br>expressing a seed-mutant version of mirt-35 that does not rescue<br>generated by microinjection into MT14533	S2E
MLC2354	nDf49, nDf50 / mIn1, II<br>lucEx1241 (mir35p::GFP_mirtron35(seed-mutant)::tbb2, myo2::mCherry)	balanced mir-35 family deletion,<br>expressing a seed-mutant version of mirt-35 that does not rescue<br>generated by microinjection into MT14533	S2E
MLC2355	nDf49, nDf50 / mIn1, II<br>lucEx1242 (mir35p::GFP_mirtron35(seed-mutant)::tbb2, myo2::mCherry)	balanced mir-35 family deletion,<br>expressing a seed-mutant version of mirt-35 that does not rescue<br>generated by microinjection into MT14533	S2E
**miR-51 family related**			
MLC1800	n4114, nDf67, IV; nDf58, X;<br>lucEx1057	mir-51 family deletion<br>rescued by a partially penetrant extrachromosomal array<br>expressing mir-54-56<br>generated by microinjection into MT17143	1C, S1D, S1E, 2B, S2E
MLC836	n4114, nDf67, IV/nT1 [qIS51] (IV,V); nDf58, X;<br>lucEx525 (mir52p::GFP_mirtron54.1::tbb2, myo2::mCherry)	balanced mir-51 family deletion,<br>expressing mirtron-51, line 1,<br>homozygous mutant line can be isolated,<br>generated by microinjection into MT17143	S2E
MLC839	n4114, nDf67, IV/nT1 [qIS51] (IV,V); nDf58, X;<br>lucEx528 (mir52p::GFP_mirtron54.1::tbb2, myo2::mCherry)	balanced mir-51 family deletion,<br>expressing mirtron-51, line 2,<br>homozygous mutant line can be isolated,<br>generated by microinjection into MT17143	S2E
MLC903	n4114, nDf67, IV/nT1 [qIS51] (IV,V); nDf58, X;<br>lucIs24 (mir52p::GFPstop_mirtron54.1::tbb2, myo2::mCherry)	balanced mir-51 family deletion,<br>expressing mirtron-51, line 3 (stop codon in GFP exon 1)<br>homozygous mutant line can be isolated,<br>generated by microinjection into MT17143, spontaneous integrant	2B, S2E
pash-1(ts)			
MLC860	mj100, I	pash-1(ts), strain SX1359 (Lehrbach et al., 2012),<br>outcrossed four times, lacking mjEx331	S1D-G, S2G, 3A, S3B, 4A, 4B, S4A, S4B
MLC881	mj100, I;<br>mjEx331 (eft-3p::pash-1::GFP::unc-54, myo-2p::mCherry::unc-54 3'UTR)	pash-1(ts), strains SX1359 (Lehrbach et al., 2012),<br>outcrossed four times	S1F
MLC1104	mj100, I;<br>lucIs20 (m35p::GFP_mirtron-35::tbb2, myo2::mCherry)	pash-1(ts)<br>expressing mirtron-35,	S3B, S3H
MLC1105	mj100, I;<br>lucIs24 (mir52p::GFPstop_mirtron54.1::tbb-2, elt2::dsRed, myo2::mCherry)	pash-1(ts),<br>expressing mirtron-51	S3B, S3H
MLC1795	mj100, I;<br>lucIs20 (m35p::GFP_mirtron-35::tbb2, myo2::mCherry),<br>lucIs24 (mir52p::GFPstop_mirtron54.1::tbb-2, elt2::dsRed, myo2::mCherry)	pash-1(ts)<br>expressing mirtron-35 and mirtron-51	S1G, S2G, 3A, 3B, S3E-H, 4A, S4A, S4B
**Auxin-inducible-degron**			
MLC1040	ieSi57 [eft-3p::TIR1::mRuby::unc-54 3'UTR + Cbr-unc-119(+)], II;<br>ieSi38 [sun-1p::TIR1::mRuby::sun-1 3'UTR + Cbr-unc-119(+)], IV	control strain expressing TIR-1 in soma & germline	1A, S1C, 3A
MLC1077	ieSi57 [eft-3p::TIR1::mRuby::unc-54 3'UTR + Cbr-unc-119(+)], II;<br>ieSi38 [sun-1p::TIR1::mRuby::sun-1 3'UTR + Cbr-unc-119(+)], IV;<br>lucIs20 (m35p::GFP_mirtron-35::tbb2, myo2::mCherry);<br>lucIs24 (mir52p::GFPstop_mirtron54.1::tbb-2, elt2::dsRed, myo2::mCherry)	control strain expressing TIR-1 in soma & germline<br>expressing mirtron-35 + mirtron-51	3B, S3E-G
MLC1065	luc71 (pash1::PASH-1::2xGGSG::3xFLAG::AID::myc), I;<br>ieSi57 [eft-3p::TIR1::mRuby::unc-54 3'UTR + Cbr-unc-119(+)], II;<br>ieSi38 [sun-1p::TIR1::mRuby::sun-1 3'UTR + Cbr-unc-119(+)], IV	endogenous pash-1 bearing C-terminal AID-tag = pash-1:AID<br>expressing TIR-1 in soma & germline	S1A, S1C
MLC1245	luc82 (myc::AID::3XFLAG::4xGGSG::drsh-1::4xGGSG::3xFLAG::AID::myc), I;<br>ieSi57 [eft-3p::TIR1::mRuby::unc-54 3'UTR + Cbr-unc-119(+)], II;<br>ieSi38 [sun-1p::TIR1::mRuby::sun-1 3'UTR + Cbr-unc-119(+)], IV	endogenous drsh-1 bearing N- and C-terminal AID-tag = drsh-1:AID<br>Expressing TIR-1 in soma & germline	S1A, S1C
MLC1726	luc71 (pash1::PASH-1::2xGGSG::3xFLAG::AID::myc),<br>luc82 (myc::AID::3XFLAG::4xGGSG::drsh-1::4xGGSG::3xFLAG::AID::myc), I;<br>ieSi57 [eft-3p::TIR1::mRuby::unc-54 3'UTR + Cbr-unc-119(+)], II;<br>ieSi38 [sun-1p::TIR1::mRuby::sun-1 3'UTR + Cbr-unc-119(+)], IV;	Endogenous drsh-1 and pash-1 bearing AID-tag<br>expressing TIR-1 in soma & germline	1A, S1C-E, 3A, S3A, S3B
MLC1727	luc71 (pash1::PASH-1::2xGGSG::3xFLAG::AID::myc),<br>luc82 (myc::AID::3XFLAG::4xGGSG::drsh-1::4xGGSG::3xFLAG::AID::myc), I;<br>ieSi57 [eft-3p::TIR1::mRuby::unc-54 3'UTR + Cbr-unc-119(+)], II;<br>ieSi38 [sun-1p::TIR1::mRuby::sun-1 3'UTR + Cbr-unc-119(+)], IV;<br>lucIs20 (m35p::GFP_mirtron-35::tbb2, myo2::mCherry);	endogenous drsh-1 and pash-1 bearing AID-tag<br>expressing TIR-1 in soma & germline<br>expressing mirtron-35	S3B-D
MLC1728	luc71 (pash1::PASH-1::2xGGSG::3xFLAG::AID::myc),<br>luc82 (myc::AID::3XFLAG::4xGGSG::drsh-1::4xGGSG::3xFLAG::AID::myc), I;<br>ieSi57 [eft-3p::TIR1::mRuby::unc-54 3'UTR + Cbr-unc-119(+)], II;<br>ieSi38 [sun-1p::TIR1::mRuby::sun-1 3'UTR + Cbr-unc-119(+)], IV;<br>lucIs24 (mir52p::GFPstop_mirtron54.1::tbb-2, elt2::dsRed, myo2::mCherry)	endogenous drsh-1 and pash-1 bearing AID-tag<br>expressing TIR-1 in soma & germline<br>expressing mirtron-51	S3B-D
MLC1729	luc71 (pash1::PASH-1::2xGGSG::3xFLAG::AID::myc),<br>luc82 (myc::AID::3XFLAG::4xGGSG::drsh-1::4xGGSG::3xFLAG::AID::myc), I;<br>ieSi57 [eft-3p::TIR1::mRuby::unc-54 3'UTR + Cbr-unc-119(+)], II;<br>ieSi38 [sun-1p::TIR1::mRuby::sun-1 3'UTR + Cbr-unc-119(+)], IV;<br>lucIs20 (m35p::GFP_mirtron-35::tbb2, myo2::mCherry);<br>lucIs24 (mir52p::GFPstop_mirtron54.1::tbb-2, elt2::dsRed, myo2::mCherry)	endogenous drsh-1 and pash-1 bearing AID-tag<br>expressing TIR-1 in soma & germline<br>expressing mirtron-35 + mirtron-51	1B, S2F, S2G, 3A, 3B, S3A, S3E-G, 4A, S4A
MLC2345	luc71 (pash1::PASH-1::2xGGSG::3xFLAG::AID::myc),<br>luc82 (myc::AID::3XFLAG::4xGGSG::drsh-1::4xGGSG::3xFLAG::AID::myc), I;<br>ieSi57 [eft-3p::TIR1::mRuby::unc-54 3'UTR + Cbr-unc-119(+)], II;<br>ieSi38 [sun-1p::TIR1::mRuby::sun-1 3'UTR + Cbr-unc-119(+)], IV;<br>lucIs24 (mir52p::GFPstop_mirtron54.1::tbb-2, elt2::dsRed, myo2::mCherry)<br>lucEx1235 (mir35p::GFP_mirtron35(seed-mutant)::tbb2, myo2::mCherry)	endogenous drsh-1 and pash-1 bearing AID-tag<br>expressing TIR-1 in soma & germline<br>expressing mirtron-51<br>expressing seed-mutant version of mirtron-35	S3C, S3D
**Other**			
MLC1867	nDf49, nDf50, II<br>lucEx1078 (mir35p::GFPstop_mirtron35::tbb2, myo3::mCherry, rgef1::BFP, gcy5::GFP)	control strain expressing gcy-5::GFP reporter<br>mir-35 family deletion is rescued by mirtron from ex-array	4B
MLC1907	pash-1(ts) (mj100), I<br>lucEx1078 (mir35p::GFPstop_mirtron35::tbb2, myo3::mCherry, rgef1::BFP, gcy5::GFP)<br>lucIs24 (mir52p::GFPstop_mirtron54.1::tbb-2, elt2::dsRed, myo2::mCherry)	pash-1(ts)<br>expressing mirtron-35 and mirtron-51<br>expressing gcy-5::GFP reporter to assess lsy-6 phenotype	4B
MLC1926	lsy-6 (ot71), V<br>lucEx1078 (mir35p::GFPstop_mirtron35::tbb2, myo3::mCherry, rgef1::BFP, gcy5::GFP)	lsy-6(0)<br>expressing gcy-5::GFP reporter to assess lsy-6 phenotype	4B

Table S1. Strains used and generated in this study. Related to STAR Methods. Name, genotype, and origin of strains as well as corresponding figures are denoted in the respective columns. Key strains are deposited with the CGC, other strains are available upon request.

Supplemental References

S1. W. J. Chung et al. Computational and experimental identification of mirtrons in Drosophila melanogaster and Caenorhabditis elegans. Genome Research. 21, 286–300 (2011).

S2. N. R. Markham, M. Zuker. DINAMelt web server for nucleic acid melting prediction. Nucleic Acids Research. 33, W577–81 (2005).

S3. M. M. Reimão-Pinto et al. Uridylation of RNA Hairpins by Tailor Confines the Emergence of MicroRNAs in Drosophila. Molecular Cell. 59, 203–216 (2015).

S4. D. Bortolamiol-Becet et al. Selective Suppression of the Splicing-Mediated MicroRNA Pathway by the Terminal Uridyltransferase Tailor. Molecular Cell. 59, 217–228 (2015).

S5. B. P. Lewis, C. B. Burge, D. P. Bartel. Conserved seed pairing, often flanked by adenosines, indicates that thousands of human genes are microRNA targets. Cell. 120, 15–20 (2005).

S6. C. H. Jan, R. C. Friedman, J. G. Ruby, D. P. Bartel. Formation, regulation and evolution of Caenorhabditis elegans 3′UTRs. Nature. 469, 97–101 (2011).

S7. M. E. Boeck et al. The time-resolved transcriptome of C. elegans. Genome Research. 26, 1441–1450 (2016).
